# A Systematic Review Comparing Animal and Human Scarring Models

**DOI:** 10.3389/fsurg.2022.711094

**Published:** 2022-04-22

**Authors:** Riyam Mistry, Mark Veres, Fadi Issa

**Affiliations:** ^1^ Nuffield Department of Surgical Sciences, University of Oxford, Oxford, United Kingdom; ^2^ John Radcliffe Hospital, Oxford University Hospitals NHS Trust, Oxford, United Kingdom

**Keywords:** scarring, review, animals, human, research models

## Abstract

**Introduction:**

A reproducible, standardised model for cutaneous scar tissue to assess therapeutics is crucial to the progress of the field. A systematic review was performed to critically evaluate scarring models in both animal and human research.

**Method:**

All studies in which cutaneous scars are modelling in animals or humans were included. Models that were focused on the wound healing process or those in humans with scars from an existing injury were excluded. Ovid Medline^®^ was searched on 25 February 2019 to perform two near identical searches; one aimed at animals and the other aimed at humans. Two reviewers independently screened the titles and abstracts for study selection. Full texts of potentially suitable studies were then obtained for analysis.

**Results:**

The animal kingdom search yielded 818 results, of which 71 were included in the review. Animals utilised included rabbits, mice, pigs, dogs and primates. Methods used for creating scar tissue included sharp excision, dermatome injury, thermal injury and injection of fibrotic substances. The search for scar assessment in humans yielded 287 results, of which 9 met the inclusion criteria. In all human studies, sharp incision was used to create scar tissue. Some studies focused on patients before or after elective surgery, including bilateral breast reduction, knee replacement or midline sternotomy.

**Discussion:**

The rabbit ear scar model was the most popular tool for scar research, although pigs produce scar tissue which most closely resembles that of humans. Immunodeficient mouse models allow for *in vivo* engraftment and study of human scar tissue, however, there are limitations relating to the systemic response to these xenografts. Factors that determine the use of animals include cost of housing requirements, genetic traceability, and ethical concerns. In humans, surgical patients are often studied for scarring responses and outcomes, but reproducibility and patient factors that impact healing can limit interpretation. Human tissue use *in vitro* may serve as a good basis to rapidly screen and assess treatments prior to clinical use, with the advantage of reduced cost and setup requirements.

## Introduction

Establishing an easily reproducible, standardised model for creating cutaneous scar tissue to assess scar treatments in both animals and humans is difficult to achieve. There is significant variation in the physiology of healing between animals, making extrapolation to humans a challenge (1). Within the Mammalia class, differences in the structure and physiology of skin are observed between species. Differences include the presence of fur, hair, sweat glands, or the panniculus carnosus (PC) (2). In humans, the PC is restricted to the platysma, the dartos, and over the palmaris brevis (2). The PC allows the skin to glide loosely over the underlying structures and can therefore contract, aiding in the wound healing process.

Pigs have been used for multiple research models in human disease due to their anatomical and physiological similarities to humans (3). Their skin also provides a very suitable model for scarring due to the physiological and anatomical similarities which include a thicker epidermis, elastic dermis, hair instead of fur, collagen structure, and an epidermis turnover rate of 30 days (4–7).

There have been multiple models used for assessing wound healing in murine models, but there are few which exist specifically for creating scar tissue (8). Difficulties in achieving scar models is due to the fast healing of murine skin, their strong contraction, presence of fur, and mobility of the subcutaneous fascial matrix facilitating faster healing (9, 10).

Within human research, the most common scarring models use patients with existing scars, often from an uncontrolled traumatic source such as flame burns. However these are limited by varying depths of injury and anatomical location influencing the scar produced.

This review assess the merits and limitations of currently reported scar creation models in both animals and humans. The focus will be on the methods reported to create scar tissue, rather than the results of the studies.

## Materials and Methods

Two near identical literature searches were performed using Medline; one focused on animal scar models and one focused on human scar models. The search was carried out with the support of the Oxford University Bodleian Library service.

All studies that utilised a specific model for assessing purposefully created scar tissue were included (Table 1). Normal, hypertrophic, and keloid scar tissue were included. Models that investigated the wound healing process and those in humans with scars from an existing injury such as a burn wound were excluded. Review articles were not included but their references were searched to identify any additional suitable papers.

**Table 1:** Inclusion and Exclusion criteria.

Inclusion criteria	Exclusion criteria
Studies involving normal scar tissue, hypertrophic scar tissue and keloid scar tissue Human studies Animal studies Purposefully created scars	Wound healing studies Scars from uncontrolled sources – such as trauma Review articles – but references searched to find suitable studies

### Information Sources

Ovid MEDLINE (1946 to February 2019).

### Additional Sources

The reference lists of review articles were examined to identify other suitable studies.

### Selection of the Studies

The titles and abstracts were independently screened by two reviewers, RM and MV to identify any potentially suitable studies. Full texts of potentially suitable studies were obtained and analysed to assess the proposed scar model.

### Search Methods for Identification of Studies – Animal Models

The following search strategy was used in Medline:

1. CICATRIX, HYPERTROPHIC/
2. KELOID/
3. (scarring or scars or scars or cicatrix or hypertrophic or keloid*).ab,ti.
4. (cutaneous or skin or tissue* or dermis or dermal or epiderm*).ab,ti.
5. 3 and 4
6. 1 or 2 or 5
7. RESEARCH/
8. (lab or laboratory or model* or remodel* or assess* or creat* or controlled or experiment*).ti.
9. (current and research*).ti.
10. bench.ti.
11. 7 or 8 or 9 or 10
12. 6 and 11
13. Limit 12 to animals
14. Limit 13 to (English language and yr = “2010-Current”).

### Search Methods for Identification of Studies – Human Models

The following search strategy was used in Medline:

1. CICATRIX, HYPERTROPHIC/
2. KELOID/
3. (scarring or scars or scars or cicatrix or hypertrophic or keloid*).ab,ti.
4. (cutaneous or skin or tissue* or dermis or dermal or epiderm*).ab,ti.
5. 3 and 4
6. 1 or 2 or 5
7. RESEARCH/
8. (lab or laboratory or model* or remodel* or assess* or creat* or controlled or experiment*).ti.
9. (current and research*).ti.
10. bench.ti.
11. 7 or 8 or 9 or 10
12. 6 and 11
13. Limit 12 to humans
14. Limit 13 to (English language and yr = “2010-Current”).

### Data Extraction and Management

Identified papers were assessed to determine the techniques used and the reproducibility of the models. The data extraction table has been deposited to the Oxford Research Archive.

### Registration with PROSPERO

The review is registered with PROSPERO under identification numbers CRD42021237692 and CRD42021233750.

## Results

### Animal Scar Studies

**Figure 1:**
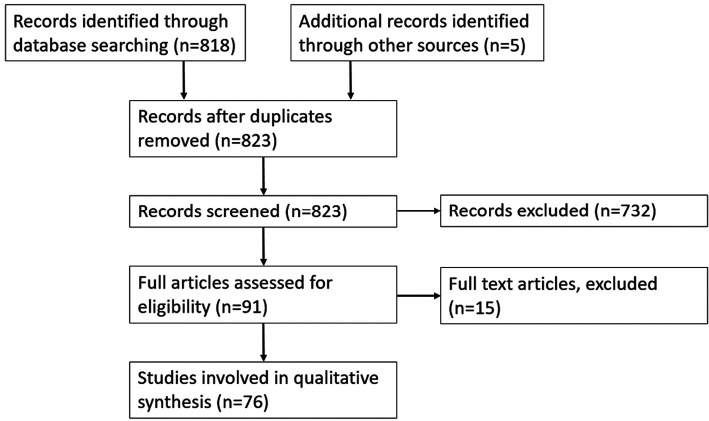
Flow diagram of records involved in analysis of animal scar models.

The search yielded 818 results in total. Both authors reviewed the titles and abstracts identifying 91 studies for further analysis including 4 review articles. Full analysis of the 91 studies identified 71 suitable studies along with an additional 5 studies that were revealed from review articles (Figure 1). Rabbits were the most used animal in scarring research, with 35 papers using them as a scar model. Murine models were second with 24 papers reporting them as a scar model. Fifteen papers reported using porcine scar models. One model used primates and one used dogs.

### Rabbit Models

All studies utilised the hypertrophic rabbit ear model first described by Morris et al 1997 (11). The technique described in all 35 studies involved creating a controlled injury on the skin of the ear resulting in a hypertrophic scar (see Table A1 in Supplementary Appendix) (12–46). A punch biopsy was the most common device used to make skin incisions, reported in 31 of the 35 studies. Four studies reported creating a circular incision but did not specify the type of device used (13–15, 34). One study compared the scar result from using a biopsy device with a controlled thermal injury (18).

Of the studies reviewed, one provided an in depth protocol for a hypertrophic ear scar model in rabbits (46). The technique involves using a 6 mm punch biopsy to make a skin excision over the ventral aspect of the ear going down to, but not including the surface of the cartilage. Exposed perichondral membrane is then dissected out, paraffin gauze placed over the wound with Tegaderm®, and gauze sutured in place over the top for 2 days. The lesion heals within 14 days with hypertrophic scars reaching a maximum size at 3–6 months post-injury (46). This then regresses over a period of 12 months after reaching its peak size. Histological analysis of the scar tissue using this model shows increased vascularity and irregularly arranged collagen fibres with a circular whorl pattern; characteristic features of hypertrophic scar tissue (46).

This method or slight variations of it (such as the size of skin excision) was used in the other 35 studies reviewed. Most commonly, the size of the wound was between 7 mm–10 mm. The smallest reported wound was 5 mm and the largest was a 20 mm circular excision (33, 42). The average amount of time the wound was left prior to animal euthanasia for wound assessment was 38.85 days. Most studies reported that the wound was fully healed at 14 days with visible scarring. Thirty studies reported creating injuries on both ears, with the remaining 5 not commenting how many ears were injured. The mean number of wounds per ear was 4.33. All studies reported using New Zealand white rabbits, with the exception of one that reported their rabbits as “adult laboratory white rabbits” (15). Outcome measures for the rabbit ear model included collagen immunhistochemical staining, vascular assessment, cytokine analysis, and the scar elevation index (SEI) of the scar. Eleven of the models reported type III collagen immunohistochemical staining as one of their outcome measures (16, 19–21, 22, 24, 26, 33, 36, 41, 43). Twelve studies involved a histological vascular assessment of scar tissue sections (12–14, 16, 19, 21, 23, 24, 33, 35, 42).

Six of the models reported quantitative polymerase chain reaction (qPCR) as an outcome tool (18, 19, 21, 26, 28, 38). Gene expression of inflammatory protein cytokines included *TNF*, *TGF-β1*, *MMP,* and *IL6*. qPCR was used in studies where a potential treatment for hypertrophic scarring was being assessed against a non-treatment control. The results were generally consistent with the intervention group showing a reduction in the expression level of these genes with the exception of *MMP-1* which was increased. Additionally, Sari et al 2017 reported higher *TGF-β1* levels in their intervention group but still reported their intervention as a potential treatment for hypertrophic scarring (19). A total of 32 studies assessed some form of intervention, with all studies reporting that this intervention resulted in an improvement in the resulting scar histologically or macroscopically. Four studies assessed ancient Chinese herbal medicine extracts (24, 27, 28, 41).

Histological analysis was performed in all studies, however Nabai et al. was the only one that compared the hypertrophic scar tissue in rabbits with human hypertrophic scar tissue (46). The height of the scar was reported as the SEI in 29 of the 35 papers, calculated by histological height of the hypertrophic scar subtracted from the histological height of the healthy skin divided by the histological height of the healthy skin (11). All 29 studies utilising the SEI showed the intervention/treatment group resulted in a lower SEI.

Many of the studies did not comment on blinding and those that did, only blinded on the histological analysis. The ear has minimal wound contraction, this is likely due to rigid adherence of the skin to the underlying cartilage. These factors combined could have an effect or influence the wound healing/scarring process and may not be representative of healing/remodelling on other body sites without underlying cartilage. Nabai et al. proposed that as the perichondral membrane is removed, an avascular surface is created increasing the risk of hypertrophic scar formation (46). The average wound harvest of 38.5 days may be a limitation as Nabai et al. left scars for up to 12 months and reported the maximum size was reached at 3–6 months (46). Rabbit scars may mature sooner or later than human scars, however animal studies requiring prolonged time frames may have cost implications.

Advantages to consider with the rabbit model include wide availability of the species, the ease of housing, strain availability, and traceability. The model is also relatively easy to reproduce and would produce hypertrophic scars with a similar appearance to that of human hypertrophic scar tissue (46). The paired nature of ears allows one rabbit to receive intervention in one ear; whilst the other ear can act as a control.

### Porcine Models

Fifteen suitable studies were identified for further analysis (see Table A2 in Supplementary Appendix) (47–59). Eleven out of the 15 studies used the Red Duroc Pig, one study used both Yorkshire white pigs and Red Duroc pigs. Liu et al. 2018 used the Bama mini pig and Jimi et al. 2017 used the Clawn mini pig (49, 54). Although likely to be a Yorkshire white pig, Chan et al. 2012 reported using “large white” pigs (58).

For the 15 papers analysed, the post-injury duration varied from 50 days at the shortest (Yun et al. 2019) to 180 days at the longest (Foubert et al. 2017) (48, 52). The range for which the wounds were reported as fully healed was from 14 to 20 days.

All the included studies involved histological assessment of the porcine scar tissue. Studies by DeBruler et al. 2018, Jimi et al. 2017 and Zhu et al. 2003 all commented on the expression of collagen in the hypertrophic scar tissue created on the pigs and compared it with human hypertrophic scar tissue. They reported collagen in mature, bundle-shaped fibres that were thick and raised; with myofibroblasts expressed in similar densities and locations to human hypertrophic scar tissue (50, 54, 60). Other similarities with human hypertrophic tissues included: skin hardening, abnormal pigmentation, flattening of the epidermis, hypervascularity, longer elastic fibres, whorl-like patterns of collagen and dysregulation of TGF-β1 (50, 54, 60). Haematoxylin and eosin (H&E), trichrome, and immunohistochemistry were performed in all but two of the studies. Most reported a reduction in the amount collagen types I and III in the control normal skin compared with the hypertrophic scars.

The Red Duroc pig was initially described in 1972 by Silverstein et al. as producing hypertrophic scars, and the model was later developed and adapted by Zhu et al. 2003 (60, 61) who described using an electric dermatome to create wounds ranging from 0.015 inches (0.381 mm) to 0.12 inches (3.048 mm) deep. After a 5 month follow up period, immunohistochemistry analysis showed similar IGF(insulin-like growth factor)-1 expression patterns to that of human hypertrophic scar tissue. However there was slightly reduced TGF-β1 expression in the porcine tissue compared to that in human hypertrophic scar tissue.

Histological analysis of the scar tissue matched that of human hypertrophic scar tissue with disorganised collagen fibres in a characteristic whorl pattern. It was proposed that Red duroc pigs were able to produce thickened scar tissue with a macroscopical and histological appearance similar to that of hypertrophic scar tissue on mid to deep dermal injury (60).

A subsequent study by Zhu et al. 2004 demonstrated the expression of IGF-1, TGF-β1 and versican (an extracellular matrix proteoglycan) was increased which is the same as in human scar tissue (62). These findings were confirmed by Gallant et al. who also identified that Red Duroc pigs produced more hypertrophic-like scarring compared to Yorkshire pigs (63, 64).

Sharp excision was reported in 3 studies; Yun et al. 2019 and Yun et al. 2012 reported creating a 3 cm × 3 cm full thickness wound using a scalpel (48, 57). Jimi et al. 2017 reported a bigger incision of 7.5 cm × 7.5 cm that was 0.15 cm deep (54). Interestingly Yun et al. 2019 and 2012 used Yorkshire pigs for their model; whilst Jimi et al. 2017 used Bama pigs (48, 54, 57).

Yun et al. 2012 reported creating 36 full thickness skin excisions on the back of the pig using a scalpel under general anaesthetic (48). After a 50-day healing period, adipose-derived stem cells (ADSC) were injected as a therapy, with full thickness biopsies taken at 10 and 23 days after treatment. These tissues underwent histological analysis and qPCR, demonstrating reduced fibroblasts and reduced expression of TGF-β1 in the ADSC group (57). Reported macroscopic scar outcomes included scar surface area calculated using photograph imaging software, colour and pliability using a durometer. The sharp excision methods described both require a degree of manual dexterity and technique to perform. The dermatome benefits from a fixed depth setting that helps to create a more controlled partial thickness injury. The red duroc pig appears to be a breed that is more prone to hypertrophic scarring compared to other breeds.

Two different methods for creating scar via a thermal injury on the pig were identified. First described by Jandera et al. 2000, a bottomless glass jug covered in waterproof tape filled with water at 82–85°C would be pressed against the skin of a pig for 10–12 s creating a contact burn (65). Two studies in the literature search reported using this method (58, 66). A variant of the Jandera model was described by Cuttle et al. 2006 (66). Their model uses the breed referred to as white pig, creating a thermal injury on the flank. A Pyrex® Schott Duran bottle with the base removed and replaced with plastic wrap is filled with water of different temperatures and applied for different times. Water at 92°C held for 15 s was optimal for achieving a deep dermal partial thickness injury (66). The pigs were followed up at 99 days post-burn. Scar tissue was on average 2.2× thicker than non-injured control tissue, with a histological appearance including collagen fibres arranged in a disorganised structure with a whorl pattern (60, 66). Electron microscopy of the porcine scar showed similarities with human hypertrophic scar tissue noting absence of rete peg grooves in both (66). An increased expression of IGF-1, Ki-67 (a proliferative nuclear marker), and cytokeratin was reported, similar to that observed in human hypertrophic scar tissue (66). The authors concluded that it their technique and methods for creating the burn wound produced a hypertrophic scar, rather than the breed of pig itself (66). Chan et al. 2012 also used the Jandera model on the backs of white Yorkshire pigs to assess the correlation between time to skin grafting and hypertrophic scarring following an acute contact burn (58). Variations included using a latex membrane at the bottom of a bottomless mug and water at 92°C held for 20 s (58). All the burn wounds underwent surgical intervention in the form of a split thickness skin graft in the study with the exception of the control burn wound that received standard burn dressings. These hot water contact burn models benefit from requiring less user skill to perform and can be done using readily available lab equipment. The depth of burn injuries is difficult to control and the temperature in this method is not kept consistent throughout the injury.

Rodriguez-Menocal et al. 2018 reported a different contact burn method to create hypertrophic scarring on red duroc pigs to assess CO_2_ and erbium-doped yttrium aluminium garnet (Er:YAG) LASERS as a treatment (47). Here, scars were created using a temperature-controlled branding iron set to 300°C and held in a vertical position under gravity for 12 s. This resulted in a 27 mm width burn wound reported to be approximately 3 mm deep. Based upon clinical experience, a similar injury in humans would result in a much more substantial depth burn. The burns were dressed with a polyurethane film and assessed weekly for scar formation. Two pigs were used in this study with twenty-seven burns being created on each pig.

The authors reported that by day 70, the scars were mature and hypertrophic in nature. Following this, the scars were treated with either CO_2_ LASER at a high or low setting; or Er:YAG at a high or low setting. Clinical assessments using a modified VSS (mVSS) and the Manchester Scar Scale (MSS) were taken on days 14, 21 and 35 with 8 mm punch biopsies taken on the last day. Er:YAG LASER treated wounds had better scores in mVSS and MSS with the best in low setting Er:YAG. The greatest amount of remodelling was observed in CO_2_ LASER treated scars. Decorin expression was greater in both LASERS on low setting and MMP-9 expression was greater in ER;YAG at low setting treated scars. The temperature-controlled branding iron has benefit over the hot water bottle model in that the temperature is kept constant throughout the injury.

The Red Duroc pig seems to have a genetic predisposition to form hypertrophic scar tissue and therefore represents a suitable model if this type of scar is desired. Those using the Yorkshire pig commented on how the healing and scarring was similar to that of normal human scarring. Advantages of using pigs include a similarity of porcine skin to human skin, a lack of fur, and the larger size of the animal allowing multiple scar sites. Several authors commented on a lack of genetic traceability of the pigs as many are obtained from the commercial farm industry.

### Murine Models

Twenty-four papers using mice or rats were identified. Of these, 15 involved creating some form of human derived scar tissue on the back of an immunosuppressed mouse kept in sterile conditions to prevent graft rejection. Four of the 15 studies involved transplanting normal human skin onto a mouse in the form of a full-thickness or split thickness skin graft (Supplementary Appendix Table A3) (67–70). The immune response is intimately involved with wound healing, therefore conclusions from studies using immunodeficient mice may be weakened (8). Four studies involved culturing of human keloid fibroblast cells and subsequently injection into the mouse (Supplementary Appendix Table A4) (71–74). Seven studies involved transplanting human keloid scar tissue onto mice (Supplementary Appendix Table A5) (75–81). Four utilised a thermal injury (Supplementary Appendix Table A6), 3 used incision and mechanical stretch (Supplementary Appendix Table A7), one used a punch biopsy model, and one a bleomycin injection model (Supplementary Appendix Table A8).

#### Studies Involving Transplantation of Human Skin onto a Mouse as a Skin Graft

A model using a Nu/Nu immunodeficient mice receiving transplanted normal human skin tissue in the form of a split thickness skin graft was described by Momtazi et al. 2013 (67).

Using discarded normal human skin after abdominoplasty, split thickness skin xenografts of set dimensions were transplanted onto mice. Skin from the dorsum of the mouse was surgically excised down to the PC to receive the xenograft. Control mice were used with split thickness skin autografts. Biopsies were taken at 30, 60, 120 and 180 days post procedure. Xenograft scars were raised, thicker, pink/red in colour with a shiny appearance compared to the control autograft scars (67). A greater average scar thickness and MSS scores (15.9 ± 0.2 and 540.9 ± 15.7 µm respectively) were reported in the xenograft scars (67). Presence of alpha smooth muscle actin (α-SMA) and reduced expression of Decorin was noted in the xenografts. The authors demonstrated that the human xenografts were alive and well for the 190-day duration with histological analysis showing an absence of rete pegs, loss of hair follicles and collagen arranged in the characteristic whorl pattern. These are all features consistent with the histological appearance of human hypertrophic scar tissue. This model was previously explored in another study by the same authors comparing scars with different graft thicknesses, concluding that human split-thickness skin grafts resulted in more hypertrophic scar tissue compared to human full-thickness skin grafts (68).

A model with similar principles was utilised by Zeplin et al. 2012 (70). The group wanted to assess the efficacy of an antifibrotic-eluting silicone gel sheet as a treatment for a burn scar. After transplanting full-thickness normal skin xenografts onto Nu/Nu mice, an additional scar was generated by burning the graft with a copper template heated to 80°C held on for 10 s. There was a reduced expression of *TGF-β1*, collagen type 1 alpha 1 (*Col1a1*), connective tissue growth factor *(Ctgf),* FGF*(Fgf)* 2, MMP-2 and 9 in the treatment group, but no comment on the effectiveness of the model to create the scar was made (70).

These models report creation of thickened scar that appears to show characteristics similar to that of human hypertrophic tissue; but the animals on which the scar develops are immunodeficient, therefore removing a significant element of the immune response during healing.

#### Studies Involving Cultured Human Scar Cells Implanted into Mice

Supp et al. 2012 implanted engineered human keloid tissue cells into the backs of immunosuppressed nu/nu mice (71). Fibroblasts and keratinocytes were extracted and cultured from human tissues, then inoculated onto bovine collagen glycosaminoglycan dermal substrates in 6 different combinations. Histological analysis showed thick, disorganised collagen bundles observed in substitutes cultured with deep and superficial keloid fibroblasts. 12 weeks after grafting, the bovine collagen biopolymer substrate was replaced by well organised human collagen (71), with no thick scars typical of human keloid scarring (71).

Wang et al. 2013 created a method of implanting a cultured human keloid fibroblast polylactic-co-glycolic acid (PLGA) scaffold on BALB/c athymic mice (72). Unlike the model by Supp et al., the engineered structures were implanted into a subcutaneous pouch within the skin of the mice, instead of an area of excised tissue. Sample collection points were 30, 60, 120 and 180 days after transplantation with the implants retrieved and fixed for immunohistochemistry and electron microscopy. The volume of tissue was noticeably larger in the keloid cultured scaffolds by day 180. Histological analysis of the keloid scaffolds showed increased immunohistochemical staining of Type I collagen, increased number of keloid fibroblasts and the characteristic whorl pattern associated with human keloid tissue (72). Under electron microscopy the authors visualised degradation of the PLGA scaffold in the control group by day 180 (72).

Lee et al. 2016 proposed the implantation of cultured human keloid scar onto Nu/J athymic mice (73). Human keloid tissue, separated into epidermal keratinocytes and dermal fibroblasts, was cultured in layers in a polyethylene ring to create a homotypic keloid skin implant. Mixed heterotypic implants were also created using normal human skin and fully homotypic human skin as a control. The implants left for a maximum of 18 weeks, with greater than 90% graft survival rate 4 weeks after implantation. The polyethylene ring would detach at 2 weeks after implantation (73). Histological analysis at 4 weeks demonstrated that collagen was more abundant in the heterotopic keloid implants than the normal tissue implants. Homotypic keloid implants showed a disrupted barrier between the epidermis and dermis, as is visualised in normal human keloid tissue. Increased expression of COL1A1, PAI-1 and urokinase receptor was reported in heterotopic keloid implants (73). Macroscopically, by 18 weeks the heterotopic and homotopic keloid implants were raised above the hosts skin (73). This model is unique in that the cells are not cultured onto a protein based scaffold, but the polyethylene ring may still influence the healing in the area. As isolated cells are used initially, this may allow for genetic manipulation of the cells to assess mechanisms and therapies.

Shang et al. 2018’s model involved injecting a concentrated suspension of human keloid fibroblasts directly into a Nu/Nu athymic mouse (74). This included a culture derived from the whole dermal keloid scar tissue that was cultured for 2 h, as well as a culture derived from dermal keloid fibroblasts only that was cultured for 24 h. The two different cultures were injected subcutaneously into the dorsum of the mice, which were subsequently euthanised 12 weeks after the injection. The group reported that by 42 days, the keloid tissue in the 2 h group was macroscopically larger than the 24 h cultured fibroblast group (74). H&E staining of the tissue from both groups showed the 2 h culture group to be more similar to human keloid scar tissue, whereas the 24 h culture group seemed to resemble normal human skin on histological appearance. The injection technique benefits from not creating an incision on the mouse and minimal disruption of the PC.

#### Studies Involving Transplanting Full Thickness Scar Tissue onto Mice

Using methods initially developed by Shetlar et al. 1985, we found three papers in our search by the same group who used a model of directly transplantation of human full thickness keloid tissue onto the back of athymic BALB/c nude mice (82). Whole human hypertrophic scar tissue was cut into 0.5 cm^3^ sections and implanted into a subcutaneous pocket on the dorsum of the mouse. The implanted scars would be retrieved at 1, 2 and 4 weeks. Interventions assessed include single injections of verapamil, verapamil + triamcinolone acetonide (TCA) and saline control directly to the scar. Administering intralesional injections of verapamil and verapamil with TCA resulted in a scar that was smaller in weight, decreased fibroblast proliferation and increased decorin expression. This same model was used to assess interferon therapy (76, 77).

Chen et al. 2017 also described a method of implanting dissected human hypertrophic scar tissue into a subcutaneous pocket on the dorsum of nude BALB/nu mice (80). The authors reported stronger decorin expression in the treatment groups, with the combination group having the strongest expression (80). Additionally the scar tissue weight was the lowest in the combination group as was the reduction in fibroblast proliferation. The authors concluded intralesional combination therapy of botulinum toxin type A (BTXA) and TCA may have therapeutic potential (80).

Fanous et al. 2019 described a very similar study utilising keloid scar tissue instead of hypertrophic scar tissue (81). For their study they implanted human keloid tissue into a subcutaneous pocket on the dorsum of nude nu/nu mice. The keloids were cut to approximately 2–3 cm^3^. One week after implantation, the implants would receive an injection of BTXA, saline as a control or TCA. Three weeks after implantation, the scars were removed and underwent histological analysis and weight assessments. They reported implants treated with BTXA or TCA had significantly smaller weights than those treated with saline (81). Blinded histological analysis revealed those treated with BTXA had a more organised collagen structure. The authors concluded BTXA may have a role as a preventative in the formation of keloid in human patients (81).

This same model was used by Qiu et al. 2015 used the same model to assess the effect of P144® an Anti-TGF-β1 topical agent (78). Seven days after implantation, one group had topical placebo applied, the other had a P144® peptide applied daily for 2 weeks. The scars were extracted and underwent histological analysis. The authors reported a reduction in the expression of collagen I and collagen III in the scars treated with P144®. They concluded that P144® may have therapeutic functions in the future but more research was needed. The subcutaneous pocket model appears to facilitate human keloid tissue growth, but the sterile housing environment combined with the immunosuppression of the mouse makes it difficult to translate to human keloid tissue.

Philandrianos et al. 2015 describes a model of suturing into place whole human keloid tissue onto the dorsums of immunosupressed nude mice (79). The keloid tissue was cut into 8 mm diameter discs using a punch biopsy and sutured into place for 4 weeks, at which point the sutures were removed. The model was used to assess 1210 nm pulsed dye LASER treatment. The keloids were harvested at 1, 2, and 3 months in all groups. The authors aimed to assess whether the LASER treatment could activate heat shock protein in human keloid scar tissue. Macroscopic and histological analysis showed no significant difference in the appearance of the scars (79).

#### Studies Using Thermal Injury to Create Scar on Mice

Ibrahim et al. 2014 developed a model to analyse scar contracture in hypertrophic tissue on immunocompetent mice (83). The thermal injury was created using a brass metal rod 8 mm in diameter heated to 100°C in boiling water for 15 min; then placing the rod on the mouse for 1 s. Three days later, the burn site was excised and a full thickness skin graft from ear skin was laid over the wound. Post-grafting, tissues were excised at days 3, 7, 9, 11, 14, 28, 70 and 168. Histological analysis demonstrated increase in vascularity, as well as macrophage and mast cells infiltration. The authors reported that the skin grafts contracted but did not disappear. Interestingly they found that the PC did not contribute to the contraction of the skin graft (83). The scars were reported as flat and initially red but later becoming pale (83).

The same model was used by Lorden et al. 2016, although here split thickness skin grafts were used (84). Prior to the graft implantation, the wound bed would be prepared with a collagen coated permeable biostable polyurethane scaffold or a polyurethane scaffold with no collagen. The authors reported that the burn wounds treated with the collagen-coated scaffold resulted in a reduced hypertrophic scar (84).

A modified version of a scald model first described by Walker and Mason et al. 1967 was reported by Lu et al. 2014 (85, 86). The thermal injury was created using a hot water bath at 100°C and placing a mouse in a plastic template that exposes 8%–10% of the total body surface area and dipping into the hot water for 8 s. Prior to thermal injury, the mice would receive clondronate liposomes subcutaneously or intraperitoneal to deplete macrophages to dampen the inflammatory response. Histological analysis at 15 days in untreated control mice showed collagen arranged in the characteristic whorl patterns (85). Quantitative PCR showed reduced TGF-β1 expression in the treated mice (85).

#### Studies Involving Mouse Skin Incision and Stretch

A novel model for wound healing and scarring was developed by Zhou et al. 2019 (9). In order to counter the contraction effect of the PC, Zhou et al. created a novel model to create scar tissue on the tail skin of rats. Full thickness tail skin excisions including the PC were made on rats using a scalpel and iris scissors at three different sizes (3 mm × 3 mm, 6 mm × 6 mm and 9 mm × 9 mm). To establish the effect of mechanical strain on scar formation in the wound site, the tails were wrapped around steel rings of set diameters of 2 cm (high strain) or 3 cm (low strain) along with a control group with no steel ring attached. Wounds were harvested at 0, 2, 6, 12 and 24 weeks post re-epithelialisation. The morpohology of the scars on the tail wounds demonstrated no wound contraction compared with control scars on the dorsum of the rats back (9). Rat tail wounds that were put under higher strain exhibited noticeably thicker and elevated scar tissue compared to the wounds under low strain (9). The wounds under no strain had the flattest scars. Histological analysis at 12 weeks of the scar tissue under strain showed signs similar to human hypertrophic scar tissue including irregular collagen fibres in a whorl like pattern. Expression patterns of TGF-β1 and α-SMA in the scar tissue placed under high strain were equivocal to the expression patters in human hypertrophic scar tissue (9). This model of creating a hypertrophic scar on a strained rat tail offers advantages over the other animals as the rat is immunocompetent, is easy to house and offers a way to minimise the effect of the PC.

A different incision and mechanical stress model was proposed by Murphy et al. 2019 (87). The authors looked to assess the effect of angiotensin type 1 receptor blocker Losartan in the resulting healed cutaneous scar. Scars were created using a scalpel to make a full skin thickness 2 cm linear incision on the dorsum of the mouse, and then sutured. Three days after the procedure, the sutures were removed and the wounds edges attached to a mechanical loading device. The device would stretch the wound by a further 4 mm every 2 days to a maximum total of 2.4 cm. At 28 days the mice were euthanised and the resulting scar excised for histological analysis. The authors reported a reduction of the scar area in those treated with losartan along with a reduction in expression of α-SMA, macrophages and collagen I fibres (87). Shan et al. also performed an incision and mechanical stretch model for creating hypertrophic scars on mice similar to the Murphy et al. model (88). They did not specify the extent to which the device stretched the wound over the ten day period. Topical naringenin was assessed as a scar treatment against non-treatment control. On day 14 the mice were euthanised and the scars excised for histology, qPCR and western blot analysis. The authors reported that naringenin inhibited fibroblast activation, suppressed inflammatory cell infiltration and reduced the expression of the inflammatory cytokines IL-1β, IL-6, TGF-β1 and TNF-α (88).

#### Studies Involving other Murine Methods

A punch biopsy method was described by Sahin et al. 2012 to assess the use of a commercial topical scar treatment (89). The scar was created by performing a full thickness punch biopsy of the skin on the dorsum of the rats and left to heal for 10 days. From this point, the topical treatment was applied. Scars were harvested on the 30th day post injury. The authors reported reduced expression of TGF-β1, fibronectin and laminin in scars treated with the topical agent. This model is not often used as the rats superior healing ability aided by the PC and the small scars that result due to its contraction.

One paper was found that described a method of injecting bleomycin, an antibiotic known to cause lung fibrosis when inhaled, into the skin to create scar tissue (90, 91). Cameron et al. 2014 described subcutaneous infusions of the antibiotic bleomycin to create hypertrophic scarring in immunocompetent mice (91). This is an adaptation of a model originally developed by Yamamoto et al. 1999 to induce sclerotic skin in mice (92). An osmotic pump sutured into a subcutaneous pocket between the muscle and the skin delivered bleomycin at a constant rate of 0.11(mu)l/hour for 28 days. Scar samples were harvested at the end of the infusion and a further set 28 days post the end of the transfusion. The histological appearance of the bleomycin mouse skin was similar to human hypertrophic scar tissue (91). The samples taken at 56 days had a significantly increased dermal thickness but a thinner epidermis than the 28-day samples.

A unique feature of this model is the lack of damage to the epidermis in creating the scar that helps to avoid the contraction created by the PC.

### Other Animal Models

One study reported using non-human primates (NHPs) and one reported using a dog in the (Supplementary Appendix Tables A8 and A9). Igarashi et al. 2015 used marmosets to analyse the effect of a pyrrole-imidazole polyamide (PIP) that targets the human TGF-β1 gene to reduce its expression (93). For this study, marmosets received a full thickness linear incision down to the PC, 2 cm in length on the abdomen. Prior to the incision, the area to be incised would receive an injection of one hundred micrograms of the PIP agent GB1101 dissolved in H_2_O with the scars harvested at 35 days. Another set of incisions received GB1101 as an ointment rubbed under the skin around the incision site prior to suturing, with the scar harvested at 42 days. For both injection and ointment GB1101, histological analysis showed a thinner epidermis and a thinner dermis resulting in a flatter scar than the control group. Although humans may be more closely related to primates, the relative difficulty in obtaining primates for research and ethical concerns in research are limitations.

Kimura et al. 2011 used the Mexican hairless dog, creating 3 cm × 3 cm full thickness skin incisions to generate hypertrophic scar (94). Histological analysis at 90 days demonstrated well-organised collagen with elastin present. Macroscopically, the scarring pattern was unique in that the dogs formed hypertrophic, hyperpigmented scars that are different to those of dogs with fur (94).

### Human Scar Studies

**Figure 2:**
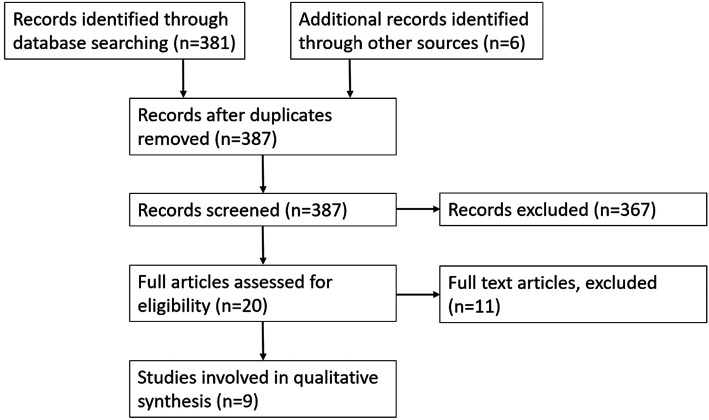
Flow diagram of records involved in analysis of human scar models.

The search yielded 381 results in total (Figure 2). Both reviewers analysed the titles and abstracts identifying 20 studies for further analysis. Full analysis of the 20 studies identified 3 suitable studies and an additional 6 studies were identified by reviewing references of review articles (Supplementary Appendix Tables A10 and A11). Reliable, easily reproducible human scar models are very rare. There are multiple models for patients that have already got a scar from a previous injury such as a burn. Very few models are present that utilise a standardised, purposefully created scar. Of the limited models that are particularly looking at scar tissue, they are typically used to assess treatments for scarring used in clinical practice.

#### *In vivo* Human Participants

A model was reported by Cruz-Korchin et al. 1996 and Niessen et al. 1998 on female patients undergoing bilateral breast reduction (95, 96). Cruz-Korchin et al. randomised 20 patients 2 weeks post bilateral breast reduction surgery to wear silicone gel sheeting on one breast (95). The patients would act as their own control and the silicone dressing would be worn for 2 months. Niessen et al. used the same scar model; in their study they included 155 patients who would wear a silicone dressing on a randomly allocated breast scars (right medial and left lateral or right lateral and left medial) for 3 months (96). Again, the patients acted as their own control. The follow up period for the Cruz-Korchin et al. study was 6 months; they reported that in breast scars left untreated, 45% developed flat scars and 55% developed hypertrophic scars. In the silicone treated group, 75% developed flat scars and 25% developed hypertrophic scars (95).

The follow up period for the Niessen et al. study was 12 months, with the authors assessing the total number of hypertrophic scars. They reported that at 3 months, 64.3% of patients had at least one hypertrophic scar which then dropped to 35.3% at 12 months (96). At 6 months, 29 patients with silicone-treated scars developed hypertrophic scars, versus 13 patients who developed hypertrophic scars on non-silicone treated control sites (*p* value = 0.006) (96). At 12 months they reported 19 hypertrophic scars in those treated with silicone and 7 in those without (*p* value = 0.02). At 3 months, the numbers were almost equal at 17 and 18 (96). However, there are several issues that make this model difficult to reproduce. There is an element of surgeon variability in the model, as well as tensions across the wounds differing depending on the volume of breast removed. The Niessen et al. study suffered from loss to follow up bias and both studies did not comment on whether patients and observers were blinded. The Cruz-Korchin study consisted mainly of Hispanic patients that have been reported to be at increased risk of hypertrophic scarring compared to Caucasian patients which may influence the findings (97).

A different scar model was utilised by Kong et al. 2004 to assess the efficacy of liquid silicone gel on pain and itch after elective total knee replacement surgery (98). The 100 patients involved in the study received surgery to one knee only. Five days after the surgery, patients were randomised to receive either silicone gel or placebo for one month. They were subsequently followed up at 3 months, 6 months, and 12 months. They reported thinner and lighters scars in those treated with silicone (silicone gel 1.5 ± 0.61 vs 1.92 ± 0.8 *p*-value = 0.004 in VSS pigmentation score) and (silicone gel 0.86 ± 0.6 vs 1.14 ± 0.75 *p*-value = 0.044 in VSS height score).

This model again relies on the same surgeon making the same cut to reduce variability. The authors reported this study was double blinded with the patients unaware of whether they were applying silicone gel or placebo. Assessors of the scar were also blinded reducing bias in the study. Placebo controls were on different patients to those having intervention. Scarring is so unique to the individual, it is difficult to assess whether the differences observed between scar in intervention vs control are accurate.

In a similar suit to the aforementioned studies, Sproat et al. 1992 used cardiac patients with established midline sternotomy scars from previous surgery (99). Fourteen patients were included in this study, one half of the scar received a TCA injection, the other half had silicone gel sheeting applied for 12 h a day for 12 weeks. Outcomes were the patients’ preference in terms of the appearance of the scar, pain, itch, and ease of the treatment. The authors reported that 11 of the 14 patients preferred silicone gel sheeting, 2 preferred the TCA injection and 1 had no preference (99).

A novel jig was developed by Dunkin et al. to create a graduated precise depth injury in the skin of healthy volunteers (100). The jig used was designed to help establish a critical depth of skin injury that would result in scar tissue formation. The authors included 113 healthy volunteers who underwent a graduated skin incision using the jig on the lateral side of the hip between the anterior superior iliac spine and the greater trochanter. Patients would be followed up weekly for 1 month, then at 6, 10, 18, 24, and 36 weeks. Outcome measures were standardised photographs (assessor blinded), a high-frequency ultrasound scanner and a dedicated image analysis software package. The jig produced a wound 51.3 ± 0.6 mm in length, at 36 weeks the mean length of 34.9 ± 1.0 mm with approximately 68% of the original wound length healing with a visible scar, the remainder without (100). The jig was designed to produce a wound with a maximum depth of 1.6 mm after previous work by the authors demonstrated that skin thickness on the lateral hip is 1.6 ± 0.1 mm (100). Using trigonometry, the authors demonstrated that the mean threshold depth on the lateral aspect of the hip that resulted in visible scar formation was 0.56 ± 0.03 mm or 33.1% of the skin thickness (100). The device is unique in that it provides a method of creating a standardised scar in a healthy human.

An interesting model was developed by Lanier et al. 2016 using the skin on the lower abdomen of patients due to undergo abdominoplasty (101). Patients would receive a series of discrete 2 cm full thickness incision on the lower abdomen under local anaesthetic. Incisions would be in parallel to each other with patients receiving up to 20, sutured, and left to heal leaving a scar. The 20 scars were used to assess a drug (unspecified by authors) in a phase 2 trial designed to help reduce scarring. The authors did not specify at what point after incision the drug was given, but one side of the abdomen was randomised to receive the drug and the other randomised to receive placebo. Scars were analysed over a 13-week period with the most lateral scars being biopsied for histological and mRNA analysis. The results from the study using that drug have not been published but the model is an interesting use of human skin that is planned to be discarded with elective abdominoplasty.

#### *In vitro* Human Scar Studies

Although the studies predominantly discussed in this review are *in vivo* human and animal models; *in vitro* scar models that use human cells are also important (Supplementary Appendix Table A11).

Ex-vivo skin cultures use human skin derived structural cells such as keratinocytes, fibroblasts, melanocytes and Langerhans cells. When cells such as keratinocytes are cultured and placed on a fresh culture media plate, they can be “wounded”. A sterile device such as a pipette tip can be used to create the wound through the cell culture. Although predominantly used in healthy normal cells, there are some culture models that specifically culture cells from hypertrophic scar tissue.

Lee et al. 2013 created a culture using human keloid tissue, specifically from the dermis of human participants with ongoing active keloid scars (102). As dermis is not usually exposed to air, the keloid tissue was cultured submerged at 37°C in a humidified atmosphere at 5% CO_2_. The keloid tissue was cut into identical spheres prior to being cultured. These spheres underwent immunohistochemical analysis which showed high levels of expression of collagen I and TGF-β1 just like in normal keloid scar tissue. Interestingly, the authors injected some of the cultured keloid sphere with TCA. They reported after injection with the steroid, the cultured keloid spheres regressed and expression of collagen I, collagen III, elastin and fibronectin was reduced just like in keloid tissue in the skin (102).

Another technique used to co-culture keratinocytes and fibroblasts and use them to form a 3D structure was reported by Chawla et al. 2018 (103). They used a collagen based gel enriched with fibroblast culture from hypertrophic scar tissue to create a 3D structure. These cultured scar tissue structures have similar α-SMA expression to that of non-cultured hypertrophic scar tissue (103).

There are various methods reported for producing a full thickness human-skin equivalent. These involve using cultured fibroblasts and keratinocytes on a collagen-based structure to form a human-skin equivalent.

A novel technique utilised by Reijnders et al. 2015 aimed to use immortalised fibroblast and keratinocyte cell lines to produce a human skin equivalent, instead of cells from fresh human tissue (104) Human telomerase reverse transcriptase (TERT) immortalised fibroblasts and keratinocytes were used to construct a human skin equivalent. The 3D construct for the skin equivalent is a bovine matrix which lacks a basement membrane, and is made up of collagen I, III, V and elastin. Fibroblasts were seeded onto this matrix and submerged for 3 weeks in culture. Keratinocytes were then seeded onto the matrix and submerged for 4 days in culture and then later cultured for 14 days exposed to air. The TERT cell human skin equivalent was compared with normal human skin and with human skin equivalent derived from primary cell culture. The authors reported that the morphology of the TERT cell engineered tissue closely resembled that of real human skin and the skin equivalent derived from primary cell culture. This includes a distinct epidermis and a fibroblast populated dermis. Further electron microscopy of the TERT cell line human skin equivalent demonstrated a well-developed stratum corneum layer made of corneosomes. The authors also showed well-developed dermosomes within the stratum granulosum, stratum spinsoum and stratum basale. A lamina lucida and lamina densa were seen on the electron microscopy images of the TERT cell line human skin equivalent suggesting formation of basement membrane. This was also confirmed with expression of basement membranes laminin 5 and collagen IV staining on immunohistochemistry.

To demonstrate if this model can be used to analyse injury and healing of human skin, the authors performed a cold injury and a burn injury to the engineered TERT cell line human skin equivalent. The epidermis was able to re-epithelialise and produce wound-healing mediators. Additionally, using the model, burn injuries would typically disrupt the basement membrane whereas in cold injuries the basement membrane would remain intact (104). The authors concluded that the human skin equivalent they have developed through immortalised cell lines could be a very useful model as it does not rely on fresh human tissues and can be relatively easily created.

## Discussion

A number of models have been reported for creating a wound in mammals, analysing the healing process, and identifying therapeutic targets for scarring (8). On review of the literature, the most popular animal model was the rabbit ear model. Rabbits are relatively inexpensive to house compared to larger animals such as pigs, while ease of reproducibility and a lack of the PC means no strong contraction of tissue. Additionally, multiple scars can be created per ear; the paired nature of ears allows for one ear to act as intervention and the other as an internal control. Pigs may be a much more suitable model for scarring studies as their skin anatomy and physiology is very similar to human tissue, such as a thick dermis and hair instead of fur. In terms of scarring models, the Red Duroc pig has been extensively investigated by Zhu et al. (60, 62, 105). Here a dermatome is used at specific depths to create a wound that subsequently scars. Further work in Red Duroc pigs has led to the suggestion that there may be a genetic element specific to the breed itself that promotes the development of thickened scar tissue (64). The contact burn technique demonstrated by Cuttle et al. highlights a simple, easily reproducible, cost-effective method of producing a burn injury. Interestingly, they used a different breed referred to as the white pig, that was able to produce hypertrophic scar tissue similar to that seen in the Red Duroc pig models. However, there are financial and ethical concerns in using a large animal model.

Murine models, have been used extensively to study the wound healing process itself, but very few have been used to produce scar tissue for analysis. The prominent PC causes contraction of wound sites, which heal quickly without scarring similar to humans. However, the transplantation of human tissue on to immunodeficient animals may result in human scars. However, the lack of a full inflammatory response in the mouse reduces the translatability of any findings. Importantly however, micer are cheaper than larger animals, easy to maintain, and have consistent genetic backgrounds. Additionally, a plethora of research tools for analysis are available for murine species.

The human bilateral breast reduction models discussed by Cruz-korchin et al. and Niessen et al. allow patients to act as their own control and are in patients already undergoing elective procedures. Limitations are the variability in the surgical techniques such that it is difficult to reproduce the exact same incisions by hand. The Northwestern abdominoplasty scar model proposed by Lanier et al. is similar in that it utilises patients undergoing an elective procedure and in this model patients act as their own controls. However, longer term outcomes cannot be assessed as the tissue is discarded when the abdominoplasty takes place. The model used to create a scar by Dunkin et al. is unique in that it is the first of its kind to utilise a device that creates a standardised incision in the skin as the blade is on jig that creates standardised incisions in depth and length. All human studies are limited by issues with compliance of treatment, loss to follow up and difficulties retrieving a biopsy for scientific analysis.

While in vitro models are useful, they cannot recreate the complex interactions in a whole animal system including those related to the immune response, nor can the normal tissue movements, such as movement of skin over a joint, be recreated. Nevertheless these are useful for initial therapeutic studies.

Despite there being multiple models to create wounds and analyse them in animals, few exist specifically looking at scar tissue. Although porcine models are the closest animal skin to human skin, their high cost, genetic variability and large size makes them difficult to work with. The mouse model using grafted human skin is promising as a future model as the results may be more translatable to humans. However, the lack of a fully functional immune system may impact the scarring process. Despite these limitations, it may emerge as a very reliable and relevant model. In human studies, the jig developed by Dunkin et al. is the most effective method for producing standardised scars, and is therefore likely to be the best for phase II/III clinical studies.

## Data Availability

The original contributions presented in the study are included in the article/Supplementary Material, further inquiries can be directed to the corresponding author/s.

## Conclusion

Scarring remains a difficult condition to treat, with many treatments utilised on a background of low-quality evidence. In order to assess a scar treatment, a robust model to create standardized scar tissue aids in producing the best quality evidence. Despite problematic scarring having an impact globally, research into scar treatments is limited and often assessed in human models with pre-existing non-standardized scars. In this review we have discussed the merits and limitations of currently reported scar creation models in both animals and humans. By reviewing and summarizing what is available, we hope to provide a reference for clinicians and scientists that are considering assessing a treatment they have developed.
