# Supplementary material for: A Systematic Review Comparing Animal and Human Scarring Models
Source: Front Surg. 2022 Apr 22;9:711094. doi: 10.3389/fsurg.2022.711094 (PMC9073696; doi:10.3389/fsurg.2022.711094)
Supplement: Supplementary file 1 [file DataSheet1.docx]

**Mistry R, Veres M and Issa F (2022) A Systematic Review Comparing Animal and Human Scarring Models. Front. Surg. 9:711094. doi: 10.3389/fsurg.2022.711094**

**Supplementary Appendix**

# APPENDIX

TABLE A1 | Results from rabbit models.

| Studies | Intervention | Scar Method | Punch Biopsy size (mm) | Data collection points (days) | Outcomes | Results | Authors comments |
| --- | --- | --- | --- | --- | --- | --- | --- |
| Caliskan | Intralesional injection | Punch | 8 | 30, 60 | Histological analysis | SEI - TCA 1.41 ± 0.17 | TCA and 5-Fu are |
| et al. ([22](#_bookmark20)) | TCA vs 5-FU vs BTXA | biopsy to |  |  |  | vs 5-FU 1.02 ± 0.22 | effective monotherapies |
|  |  | to both |  |  |  | vs BTA 0.98 ± 0.3 | for HS, BTXA no effect |
|  |  | ears |  |  |  | 5-Fu reduces  ﬁbroblast count. | on established HTS |
| Chavez- | Indoleamine 2,3- | Punch | 8 | 35 | Histological analysis, | SEI – Transduced | IDO transduced |
| Munoz et al.  ([37](#_bookmark34)) | dioxygenase (IDO)  transduced ﬁbroblasts | biopsy to  to both |  |  | western blot | IDO 1.4 ± 0.04 vs  non-transduced IDO | ﬁbroblast skin  substitutes provide a |
|  | vs non-transduced  ﬁbroblasts vs non- | ears |  |  |  | 2.3 ± 0.25 vs control  2.3 ± 0.22 | wound coverage that  results in a better scar |
|  | treated (control) |  |  |  |  |  |  |
| Demir et al. | Intralesional Enalapril | Punch | 6 | 28, 40 | Histological analysis of | SEI - Lanapril 1.46 ± | Better scar appearance |
| ([16](#_bookmark14)) | vs Candesartan vs  TCA vs non-treatment | biopsy to  to both |  |  | arrangement of collage  ﬁbres | 0.29 vs candesartan  1.62 ± 0.35 vs steroid | macroscopically and  microscopically in those |
|  | control | ears |  |  |  | 1.26 ± 0.15 vs control | treated with ACE-I and |
|  |  |  |  |  |  | 1.97 ± 0.35 | steroid |
| Diao et al. | Intradermal 0.02% | Punch | 10 | 16, 23, 45 | Histological analysis, qPCR, | Decreased Collagen | Histone deacetylase |
| ([34](#_bookmark31)) | trichostatin vs | biopsy to |  |  | western blot | 1, ﬁbronectin in | inhibitors may be an |
|  | intradermal saline | to both |  |  |  | treatment group | effective therapeutic |
|  | (control) | ears |  |  |  | SEI – 0.02% | strategy for HTS |
|  |  |  |  |  |  | trichostatin 1.45 ± |  |
|  |  |  |  |  |  | 0.09 vs control |  |
|  |  |  |  |  |  | 2.07 ± 0.10 |  |
| Fang et al. | Topical opuntia extra | Punch | 7 | 22, 39, 54 | Histological analysis, qPCR, | Improve histological | Opuntia extract |
| ([24](#_bookmark22)) | vs topical saline | biopsy to |  |  | scar thickness assessment | appearance and | decreases the formation |
|  | control | to both |  |  |  | collagen deposition, | of HTS |
|  |  | ears |  |  |  | increased MMP-1 |  |
|  |  |  |  |  |  | expression |  |
| Friedrich | Burn injury + | Punch | 10 | 1 h, 3, 28, | Histological analysis, qPCR | SEI - Surgical | Thermal injury via brass |
| et al. ([18](#_bookmark15)) | debridement vs | biopsy to |  | 35 |  | (control)1.34 ± vs | rod for 20 s produces |
|  | surgical excision | to both |  |  |  | Burn wound 0.22 | HTS |
|  | (control) | ears |  |  |  | 1.63 ± 0.37, no |  |
|  |  |  |  |  |  | difference in TNF-α on qPCR |  |
| Gisquet et al. | Intradermal tacrolimus | Punch | 10 | 14, 28, 60 | Histological analysis, | SEI - Intradermal | Intradermal tacrolimus |
| ([44](#_bookmark41)) | vs non-treatment | biopsy to |  |  | Bimodal spectroscopy, | tacrolimus 1.5 ± 1.5 | prevents HTS, bimodal |
|  | injection (control) | to both |  |  |  | vs Control 3.1 ± 1.7 | spectroscopy may have |
|  |  | ears |  |  |  |  | a role in characterising |
|  |  |  |  |  |  |  | physiopathology |
| Gong et al. | Cultured HTS | Punch | 7 | 28 | Histological analysis and | Down regulation of | RHE inhibits HTS |
| ([21](#_bookmark19)) | ﬁbroblasts cultured | biopsy to |  |  | qPCR | cyclinD1, cyclin- | ﬁbroblast proliferation |
|  | cells treated with RHE | to both |  |  |  | dependent kinase 4, |  |
|  |  | ears |  |  |  | proliferating cell |  |
|  |  |  |  |  |  | nuclear antigen |  |
| Gong et al. ([17](#_bookmark16)) | RHE intradermal  injections vs TCS vs saline control | Punch  biopsy to to both | 7 | 28, 47 | TEM, ﬂow cytometry | Increased apoptosis  of HTS ﬁbroblasts on electron microscopy | RHE inhibits hypetrophic scar  ﬁbroblast proliferation |
|  |  | ears |  |  |  |  |  |
| Hartwell et al. | IDO secreting | Punch | 6 | 1, 3, 5, 7, | Histological analysis, IHC, | Reduced contraction, | Application for a gel |
| ([29](#_bookmark27)) | ﬁbroblast scaffold vs  acellular scaffold | biopsy to  to both |  | 20, 35 | immunoﬂuoresence,  macroscopic analysis | reduced  inﬂammatory | scaffold to facilitate  healing |
|  |  | ears |  |  |  | proteins |  |
|  |  |  |  |  |  | SEI - Gel 1.24 ± 0.05 |  |
|  |  |  |  |  |  | vs Gel IDO 1.25 ± |  |
|  |  |  |  |  |  | 0.03 |  |
|  |  |  |  |  |  |  | (*continued*) |

TABLE A1 | Continued

| Studies | Intervention | Scar Method | Punch Biopsy size (mm) | Data collection points (days) | Outcomes | Results | Authors comments |
| --- | --- | --- | --- | --- | --- | --- | --- |
| Jia et al. ([40](#_bookmark37)) | Topical silicone gel vs | Punch | 10 | 35 | Histological analysis, wound | SEI - Given as a | Silicone gel is as |
|  | silicone gel + silver vs | biopsy to |  |  | size assessment | chart, silicone gel | effective as SGS at |
|  | untreated control | to both |  |  |  | and silicone gel + | reducing HTS |
|  |  | ears |  |  |  | silver both had lower |  |
|  |  |  |  |  |  | SEI |  |
| Ko et al. ([38](#_bookmark35)) | Intralesion inection of | Punch | 7 | 15, 21, 35 | Histological analysis, qPCR | No signiﬁcant | Statins reduce HTS |
|  | simvastatin vs | biopsy to |  |  |  | differences in SEI | formation via inhibition |
|  | lovastatin vs | to both |  |  |  | Report simvastatin, | of CTGF |
|  | pravastatin all at high, | ears |  |  |  | lovastatin, |  |
|  | medium or low dose |  |  |  |  | pravastatin reduce |  |
|  | (as control) |  |  |  |  | SEI by 21.9%, 25.8% |  |
|  |  |  |  |  |  | and 22.8% |  |
|  |  |  |  |  |  | respectively |  |
|  |  |  |  |  |  | Reduced CTGF |  |
|  |  |  |  |  |  | expression in low |  |
|  |  |  |  |  |  | dose statin |  |
| Liu et al. ([30](#_bookmark28)) | Intra-arterial delivery | Punch | 9 | 3, 10, 14, | Histological analysis for | Treatment down | Wild-type MSC |
|  | of control MSC vs p53  MSC vs no treatment control | biopsy to  to both ears |  | 21, | inﬂammatory proteins and  mesenchymal stem cell presence | regulated α-SMA, TGF-β1  SEI - Given as a | engraftment could  inhibit HTS in a p53- dependent manner |
|  |  |  |  |  |  | chart, SEI lower in |  |
|  |  |  |  |  |  | treatment group |  |
| Liu et al. ([20](#_bookmark18)) | Intralesional BTXA vs | Punch | 7 | Daily for 60 | Histological analysis of | SEI - Control 2.368 ± | Botulinum toxin-A |
|  | TCA vs no treatment | biopsy to |  | days | collagen | 0.1986 vs botulinum | improves appearance of |
|  | control | to both |  |  |  | toxin A | HTS, suppresses |
|  |  | ears |  |  |  | 1.431 ± 0.0977 vs  Triamcinalone | collagen deposition and  ﬁbroblast proliferation |
|  |  |  |  |  |  | 1.630 ± 0.0768 |  |
| Menezes | Tacrolimus ointment | Punch | 10 | 30 | Histological analysis | Scar thickness in | Tacrolimus ointment |
| et al. ([13](#_bookmark11)) | vs Vaseline control | biopsy to |  |  |  | Tacrolimus group | helps to produce a |
|  |  | to both |  |  |  | 656.69 ± 226.94 µm | thinner scar |
|  |  | ears |  |  |  | vs control 929.66 ± |  |
|  |  |  |  |  |  | 505.25 µm |  |
| Nabai et al. | Biopsy device to | Punch | 10 |  | Protocol only |  | Description of rabbit ear |
| ([46](#_bookmark43)) | create HTS | biopsy to |  |  |  |  | HTS model |
|  |  | to both |  |  |  |  |  |
|  |  | ears |  |  |  |  |  |
| Rahmani- | Topical stratiﬁn vs | Punch | 8 | 5, 20, 28 | Histological, | Increased MMP-1 in | Topical application of |
| Neishaboor | ASA incorporated | biopsy to |  |  | immunhistochemistry for | stratiﬁn and reduced | stratiﬁn and aspirin can |
| et al. ([45](#_bookmark42)) | CMC gel vs untreated  control | to both  ears |  |  | inﬂammatory proteins,  collagen proﬁle | collagen density  SEI - CMC gel1.79 ± | improve or prevent HTS |
|  |  |  |  |  |  | 0.19 vs ASA-CMC |  |
|  |  |  |  |  |  | gel 1.23 ± 0.07 vs  Stratiﬁn 1.15 ± 0.14 |  |
|  |  |  |  |  |  | vs Control 1.84 ± |  |
|  |  |  |  |  |  | 0.15 |  |
| Ren et al. ([35](#_bookmark32)) | Intraperitoneal | Punch | 7 | 15, 21, 28, | Histological analysis, | Type I collagen, Bcl- | Systemic endostatin |
|  | endostatin injection vs | biopsy to |  | 35 | western blot | 2 supressed | application inhibits |
|  | intraperitoneal | to both |  |  |  | SEI - Endostatin | angiogenesis reducing |
|  | injection of saline | ears |  |  |  | 1.09 ± 0.19 vs control | HTS |
|  |  |  |  |  |  | 1.36 ± 0.28 |  |
| Rha et al. ([23](#_bookmark21)) | SGS + 0.25 mg/g verapamil vs SGS + | Punch biopsy to | 6 | 17, 26, 53 | Histological analysis of  ﬁbroblast and capillary count | SEI - SGS 2.2  (control) vs SGS + | SGS containing verapamil microparticles |
|  | 2.5 mg/g verapamil vs | to both |  |  |  | 0.25 mg verapamil | at an optimum dose of |
|  | SGS+25 mg/g | ears |  |  |  | 1.3 vs SGS + 2.5 mg | 2.5 mg/g improves HTS |
|  | verapamil vs SGS |  |  |  |  | verapamil 1.2 vs | according to SEI |
|  | only (control) |  |  |  |  | SGS + 25 mg |  |
|  |  |  |  |  |  |  | (*continued*) |

TABLE A1 | Continued

| Studies | Intervention | Scar Method | Punch Biopsy size (mm) | Data collection points (days) | Outcomes | Results | Authors comments |
| --- | --- | --- | --- | --- | --- | --- | --- |
|  |  |  |  |  |  | verapamil 1.1 |  |
|  |  |  |  |  |  | Fibroblast and |  |
|  |  |  |  |  |  | capillary counts |  |
|  |  |  |  |  |  | lower in verapamil |  |
|  |  |  |  |  |  | groups |  |
| Sari et al. ([19](#_bookmark17)) | Intralesional DMSO vs | Punch | 5 | 28 | Histological analysis of | TGF-β1 expression | DMSO is an alternative |
|  | TCA vs saline control | biopsy to |  |  | epithelial thickness, collagen | greater in DMSO | to steroid in the |
|  |  | to both |  |  | and vascularity, | treated | treatment of HTS |
|  |  | ears |  |  | inﬂammatory gene qPCR |  |  |
| Song et al. | Intralesional Usnic | Punch | 10 | 23, 35 | Histological analysis of | Reduced CD31 | Usnic acid inhibits HTS |
| ([14](#_bookmark12)) | acid vs TCA control | biopsy to |  |  | inﬂammatory markers | staining, improved | formation |
|  |  | to both |  |  |  | collagen appearance |  |
|  |  | ears |  |  |  | SEI – Usnic acid |  |
|  |  |  |  |  |  | group 1.76 ± 0.31 |  |
|  |  |  |  |  |  | treated vs TCA |  |
|  |  |  |  |  |  | control2.85 ± 0.82 |  |
| Tark et al. | Intradermal injection | Punch | 8 | 21, 35 | Histological analysis of | Reduced MMP, | Ginsenoside Rb1 |
| ([28](#_bookmark26)) | ginsenoside Rb1  0.07 mg vs 0.28 mg | biopsy to  to both |  |  | inﬂammatory proteins and  collagen proﬁle, qPCR | TIMP1, α-SMA and  TGF-β1 | suppresses HTS in  rabbit ear model |
|  | vs 0.56 mg vs saline | ears |  |  |  | SEI - Control 4.22 ± |  |
|  | control |  |  |  |  | 0.63 vs ginsenoside |  |
|  |  |  |  |  |  | 0.07 mg |  |
|  |  |  |  |  |  | 3.40 ± 0.32 vs |  |
|  |  |  |  |  |  | 0.28 mg |  |
|  |  |  |  |  |  | 3.09 ± 0.24 vs |  |
|  |  |  |  |  |  | 0.56 mg |  |
|  |  |  |  |  |  | 1.21 ± 0.06 |  |
| Tollefsen | SGS vs Paper tape vs | Punch | 6 | 7, 14, 28, 44 | Histological analysis, | SEI - Paper tape | Paper tape and SGS |
| et al. ([39](#_bookmark36)) | Untreated (control) | biopsy to |  |  | photographic analysis | 1.32 ± 0.2 vs SGS | show equal |
|  |  | to both |  |  |  | 1.41 ± 0.18 vs control | effectiveness in |
|  |  | ears |  |  |  | 1.35 ± 0.23 | prevention of HTS on |
|  |  |  |  |  |  |  | visual analysis, but no |
|  |  |  |  |  |  |  | difference histologically |
| Tunca et al. | Cryotherapy | Punch | 8 | 16 & 28 | Histological analysis | SEI - Excision group | Cryotherapy as an |
| ([12](#_bookmark10)) | compared with | biopsy to |  |  |  | 1.52 ± 0.4 vs | alternative to making |
|  | surgical removal of | to both |  |  |  | Cryogroup | surgical removal of skin |
|  | skin | ears |  |  |  | 1.63 ± 0.5 |  |
| Uzun et al. | Early oral enalapril vs | Punch | 5 | 14, 28, 40 | Histological analysis | Improved collagen | Earl oral administration |
| ([33](#_bookmark30)) | later oral enalapril vs | biopsy to |  |  |  | proﬁle in treatment | of enalapril after dermal |
|  | intralesional steroid | to both |  |  |  | group | injury reduce HTS |
|  | injection vs no | ears |  |  |  | SEI - Early elanpril |  |
|  | treatment control |  |  |  |  | 1.3 vs late elanapril |  |
|  |  |  |  |  |  | 1.56 vs steroid group |  |
|  |  |  |  |  |  | 1.25 vs control 1.98 |  |
| Wang et al. | ASMq 400 vs 800 vs | Punch | 7 | 15, 40, | Histological analysis | Reduced collagen, | Orally administered |
| ([27](#_bookmark25)) | 1,200 mg/kg | biopsy to |  |  |  | pro-collagen in | ASMq improves the |
|  | bodyweight vs saline | to both |  |  |  | treatment | appearance of HTS |
|  | control | ears |  |  |  | SEI - Utilised but |  |
|  |  |  |  |  |  | exact ﬁgures not |  |
|  |  |  |  |  |  | given, lower in all |  |
|  |  |  |  |  |  | treatment grous |  |
| Wang et al. | Intradermal TSG-6 | Punch | 7 | 21, 28, 42 | TNFα stimulated gene 6 | TSG-6 lower levels if | Immediate dose of |
| ([26](#_bookmark24)) | injections vs Saline | biopsy to |  |  | protein (TSG-6). Histological | IL-1β, IL-6, TNF-α, | TSG-6 during healing |
|  | control | to both |  |  | analysis, qPCR, collagen | reduced collagen | can reduce the severity |
|  |  | ears |  |  | proﬁle | SEI - Day 21 Control | of HTS |
|  |  |  |  |  |  | 1.13 ± 0.09 vs TSG-6 |  |
|  |  |  |  |  |  | 1.31 ± 0.11 |  |
|  |  |  |  |  |  |  | (*continued*) |

TABLE A1 | Continued

| Studies | Intervention | Scar Method | Punch Biopsy size (mm) | Data collection points (days) | Outcomes | Results | Authors comments |
| --- | --- | --- | --- | --- | --- | --- | --- |
|  |  |  |  |  |  | Day 28 Control |  |
|  |  |  |  |  |  | 1.29 ± 0.15 vs TSG-6 |  |
|  |  |  |  |  |  | 1.92 ± 0.18 |  |
|  |  |  |  |  |  | Day 42 Control |  |
|  |  |  |  |  |  | 1.34 ± 0.18 vs TSG- |  |
|  |  |  |  |  |  | 62.93 ± 0.38 |  |
| Wang et al. | RHE intralesional | Punch | 11 | 25, 30 | Histological analysis, qPCR | Fewer collagen | RHE reduces formation |
| ([106](#_bookmark103)) | injection vs saline | biopsy to |  |  | of VEGF and tissue inhibitor | ﬁbres, smoother in | of HTS |
|  | control | to both |  |  | of metalloproteinase -1 | treatment group, |  |
|  |  | ears |  |  |  | Reduced protein |  |
|  |  |  |  |  |  | expression of VEGF |  |
|  |  |  |  |  |  | and TIMP-1 |  |
|  |  |  |  |  |  | SEI – RHE 1.37 ± |  |
|  |  |  |  |  |  | 0.21 vs control |  |
|  |  |  |  |  |  | 2.65 ± 0.21 |  |
| Wei et al. ([43](#_bookmark40)) | Topical OA 2.5% vs | Punch | 7 | 28 | Histological analysis, | TGF-β1, MMP-1 | OA suppresses HTS in |
|  | 5% vs 10% vs | biopsy to |  |  | Immunohistochemistry of | collagen I and III | the rabbit ear model |
|  | untreated control | to both |  |  | inﬂammatory proteins | SEI - OA 2.5% |  |
|  |  | ears |  |  |  | 2.63 ± 0.20 vs OA |  |
|  |  |  |  |  |  | 5% 2.12 ± 0.32 vs |  |
|  |  |  |  |  |  | OA 10% 1.87 ± 0.24 |  |
|  |  |  |  |  |  | vs Control 3.09 ± |  |
|  |  |  |  |  |  | 0.32 |  |
| Wo et al. ([31](#_bookmark29)) | Topical EG loaded | Punch | 10 | 1, 30 | Western blot and | SEI - Given as a | EG as a successful |
|  | with 5-FU vs EG | biopsy to |  |  | histological analysis | chart, SEI lower in | delivery mechanism of |
|  | loaded with saline | to both |  |  |  | treatment group | 5-Fu to a wound |
|  | control | ears |  |  |  |  |  |
| Wu et al. ([41](#_bookmark38)) | EO from ligusticum | Punch | 7 | 22, 28 | Histological analysis for | SEI - 5% essential oil | EO probably becomes |
|  | chuanxiong at 5% vs | biopsy to |  |  | inﬂammatory proteins | (EO)2.95 ± 0.33 vs | an effective cure for |
|  | 10% vs 20% vs  topical contractubex^®^ | to both  ears |  |  |  | 10% EO 2.49 ± 0.27  vs 20% EO 2.07 ± | human HTS |
|  | vs untreated (control) |  |  |  |  | 0.25 |  |
|  |  |  |  |  |  | Control 3.48 ± 0.34 |  |
| Yagmur et al. | Denervated skin vs | Punch | 20 | 14, 28 | Histological analysis | SEI - Denervated | Surgically denervated |
| ([42](#_bookmark39)) | Innervated skin | biopsy to |  |  |  | 1.26 ± 0.22 vs | skin results in reduced |
|  | (Control) | to both |  |  |  | Control 1.6 ± 0.34 | scarring |
|  |  | ears |  |  |  |  |  |
| Zhang et al. | Topical LEO 2.5% vs | Punch | 10 | 28, 56 | Histological analysis, ELISA, | Collagen I, III | LEO reduces HTS by |
| ([36](#_bookmark33)) | 5% vs 10% vs | biopsy to |  |  | qPCR | reduced in LEO | inhibiting hypertrophic |
|  | liposome without EO | to both |  |  |  | group | ﬁbroblasts and inducing |
|  | (control) | ears |  |  |  | SEI - LEO 2.5% | their apoptosis |
|  |  |  |  |  |  | 2.39 ± 0.21 vs LEO |  |
|  |  |  |  |  |  | 5% 1.96 ± 0.20 vs |  |
|  |  |  |  |  |  | LEO 10% 1.34 ± 0.11 |  |
|  |  |  |  |  |  | vs control 2.74 ± 0.24 |  |
| Zhang et al. | Intralsional injection | Punch | 10 | 14, 21, 28, | Adipose derived stem cells. | SEI - ADSC 1.08 ± | Adipose derived stem |
| ([25](#_bookmark23)) | ADSC vs ADSC | biopsy to |  | 35 | Histological analysis | 0.05 vs DMEM | cells (ADSC) can |
|  | conditioned medium | to both |  |  |  | 1.93 ± 0.09, | suppress HTS |
|  | (CM) vs DMEM vs | ears |  |  |  | ADSC-CM 1.33 ± |  |
|  | untreated (control) |  |  |  |  | 0.10 vs DMEM |  |
|  |  |  |  |  |  | 1.97 ± 0.11, |  |
|  |  |  |  |  |  | Control1.90 ± 0.12 vs |  |
|  |  |  |  |  |  | DMEM 1.94 ± 0.06 |  |
| Zhao et al. | High-ESWT | Punch | 15 | 1, 4, 7, 10, | Histological analysis of | Improved collagen | Extra-coropreal shock |
| ([15](#_bookmark13)) | (0.2 mJmm^2^) vs Low- | biopsy to |  | 14, 21, 28, | arrangement of collage | appearance | wave therapy |
|  | ESWT (0.1 mJ/mm^2)^ | to both |  | 35 | ﬁbres | SEI – low-ESWT | suppresses hypertrophy |
|  |  | ears |  |  |  | 2.32 ± 0.15 vs high- | scar formation |

(*continued*)

TABLE A1 | Continued

Studies Intervention Scar Method

Punch Biopsy size (mm)

Data collection points (days)

Outcomes Results Authors comments

vs sham ESWT (control)

ESWT 2.34 ± 028 vs

sham ESWT control 2.71 ± 0.2

*Abbreviations: 5-FU, 5- ﬂuorouracil; α-SMA, alpha smooth muscle actin; ACE-I, Angiotensin converting enzyme inhibitor; ADSC, Adiposed derived stem cells; ASA, Acetysalicylic acid; ASMq, Abnormal savda munziq; Bcl2, B-cell lymphoma 2 protein; BTXA, Botulinum toxin A; CMC, carboxymethyl cellulose; CTGF, connective tissue growth factor; DMEM, Dulbeccos modiﬁed eagle medium; DMSO, Dimethylsulfoxide; ELISA, enzyme linked immunosorbent assay; EO, essential oils; ESWT, Extra-corporeal shockwave therapy; HTS, hypertrophic scar; IDO, Indoleamine 2,3-dioxygenase; LEO, Liposome-eneveloped essential oil; MMP, matrix metalloproteinases; MSC, mesenchymal stem cell; qPCR, Quantitative polymerase chain reaction; RHE, recombinant human endostatin; SEI, scar elevation index; TCA, triamcinolone acetonide; TEM, Transmission electron microscopy; TGF, Transforming growth factor; TIMP, tissue inhibitor of metalloproteinases; TNF-α, tumour necrosis factor alpha; TSG-6, TNFα stimulated gene 6 protein; VEGF, vascular endothelial growth factor.*

TABLE A2 | Results from pig models.

| Study | Pig Breed | Scar Method | Wound number | Data collection point (days) | Intervention | Outcomes | Results | Authors comments |
| --- | --- | --- | --- | --- | --- | --- | --- | --- |
| Bailey et al. | Red | Contact burn, | 4 on each | 7, 28, 56, | PDL vs CO_2_ | Photography, Erythema | Laser | PDL ad CO_2_ laser |
| ([51](#_bookmark48)) | Duroc | 1″ × 1″, 200°C, | pig, 8 pigs | 84, 112 | laser vs PDL | via image software, Scar | intervention | therapy within 1 |
|  |  | 40 s, 3 lbs of | total |  | and CO_2_ laser | biomechanics, | resulting in less | month of auto |
|  |  | pressure, burn |  |  | vs untreated | Histology, Scar | scar contraction | STSG helps to |
|  |  | eschar then |  |  | control 28 | roughness (imaging | compared to | reduced scar |
|  |  | excised and |  |  | days post | software), | control, laser | contraction |
|  |  | covered with |  |  | injury | immunohistochemistry, | treated scars |  |
|  |  | STSG autograft |  |  |  | qPCR | were redder, |  |
|  |  |  |  |  |  |  | CO_2_ Laser scars |  |
|  |  |  |  |  |  |  | were smoother, |  |
|  |  |  |  |  |  |  | Co_2_ laser scar |  |
|  |  |  |  |  |  |  | strongest |  |
| Blackstone | Red | Zimmer^®^ | 2 burn | 0, 10, 28, | Scar formation | TEWL, scar erythema, | 2 fold greater | Burn scars |
| et al. ([53](#_bookmark50)) | Duroc | dermatome 5 × | wounds on | 90, 150 | with | scar contraction, | TEWL in burn | produce hairless, |
|  |  | 5 cm 0.06″ | each pig, 1 |  | dermatome vs | histological analysis, | group, greater | hyper/ |
|  |  | deep or 0.075″ | deeper and |  | burn | scar biomechanics, | contraction in | hypopigemented, |
|  |  | deep, Contact | 1 shallower |  |  | qPCR | burn group, scar | thicker, weaker, |
|  |  | burn, 1″ × 1″, | dermatome |  |  |  | thickness | less elastic scars |
|  |  | 200°C, 40 s, | injury on |  |  |  | greater in burn | that are more |
|  |  | 3 lbs of | each pig, 4 |  |  |  | group, stiffer and | similar to human |
|  |  | pressure | pigs total |  |  |  | weaker, TGF-β1 | HTS compared to |
|  |  |  |  |  |  |  | expression | dermatomal injury. |
|  |  |  |  |  |  |  | greater in burn |  |
|  |  |  |  |  |  |  | scar |  |
| Carney | Red | Dermatome 4″ | 2 on each | 70, 84, 126 | Automated | VSS, qPCR, Elastin | VSS pliability | Pressure treatment |
| et al. ([55](#_bookmark52)) | Duroc | × 4″, 0.09″ deep | pig, 2 pigs | (pressure | pressure | protein, histological | score greater in | results in higher |
|  |  |  | total | applied from | delivery to one | analysis | treatment group, | protein level |
|  |  |  |  | day 70 for | scar 30 mmHg |  | decrease in | expression of |
|  |  |  |  | 14 days) | vs control |  | elastin transcript, | elastin compared |
|  |  |  |  |  |  |  | elastin protein | to control, |
|  |  |  |  |  |  |  | and staining | correlated with |
|  |  |  |  |  |  |  | greater in | VSS scores |
|  |  |  |  |  |  |  | treatment group | clinically |
| Chan et al. | Large | Contact burn, | 4 burns | 0, 99 | Burn dressing | VSS, histological | Wounds grafted | Early grafting is |
| ([58](#_bookmark55)) | White Pig | bottomless | approx. |  | only vs STSG | analysis, α-SMA level, | at day 3 post- | associated with a |
|  |  | mug 300 ml | 5 cm in |  | day 3 post- | microbiological analysis | burn had the | better histological |
|  |  | water at 92°C | diameter on |  | burn, STSG |  | lowest α-SMA, | and clinical scar |
|  |  | applied for | each pig, 5 |  | day 14 post- |  | VSS score on | outcome, infection |
|  |  | 20 ss, set | pigs total |  | burn, STSG |  | day 3 grafts | may contribute to a |
|  |  | pressure |  |  |  |  | closer to normal |  |
|  |  |  |  |  |  |  |  | (*continued*) |

TABLE A2 | Continued

| Study | Pig Breed | Scar Method | Wound number | Data collection point (days) | Intervention | Outcomes | Results | Authors comments |
| --- | --- | --- | --- | --- | --- | --- | --- | --- |
|  |  |  |  |  | day 21 post- |  | skin, degree of | greater degree of |
|  |  |  |  |  | burn |  | ﬁbrosis greater in | ﬁbrosis |
|  |  |  |  |  |  |  | wounds with |  |
|  |  |  |  |  |  |  | positive bacterial |  |
|  |  |  |  |  |  |  | culture |  |
| DeBruler | Red | Contact burn, | 8 per pig, 32 | 7, 28, 63, | STSG | Photography, TEWL, | Meshed grafts | Thick graft scars |
| et al. ([50](#_bookmark47)) | Duroc | 1″ × 1″, 200 °C, | total (4 pigs | 119 | autograft | Immunohistochemistry, | greater TEWL, | decreased |
|  |  | 40 s, 3 lbs of  pressure | used) |  | 0.026″ or  0.058″ thick, | Gene expression, scar  biomechanics | thicker grafts  less deep scar, | contraction,  reduced scar |
|  |  |  |  |  | thinner grafts |  | thick graft scar | depth, mesh on |
|  |  |  |  |  | meshed |  | greater | thin grafts did not |
|  |  |  |  |  |  |  | biomechanical | affect the scarring. |
|  |  |  |  |  |  |  | strength, thick |  |
|  |  |  |  |  |  |  | graft reduced |  |
|  |  |  |  |  |  |  | TGF-β1 |  |
|  |  |  |  |  |  |  | expression |  |
| Engrav | 3× Red | Padgett^®^ | 5 of each | 7, 14, 21, 84 | Comparison of | qPCR, Porcine | 11 collagen | Collagen I, II, IV, V, |
| et al. ([59](#_bookmark56)) | Duroc | dermatome | depth per |  | collagen | GeneChip^®^, | genes and 7 | VI, VII, XIV, XVI are |
|  | and 3× | 7 cm × 7 cm, | pig, 6 pigs |  | genes |  | collagen types | involved in the |
|  | White Yorkshire | 10, depth 0.02″ and 0.06″ | total |  | expressed porcine HTS |  | identiﬁed in human and | process of  ﬁbroproliferative |
|  |  |  |  |  | with that of |  | duroc pig | scarring. |
|  |  |  |  |  | human HTS |  | hypertrophic |  |
|  |  |  |  |  |  |  | tissue |  |
| Foubert | Red | Electric | 4 on each | 14, 56, 182 | Autologous | Photography + imaging | Treatment group | Delivery of ADRC’s |
| et al. ([52](#_bookmark49)) | Duroc | dermatome, | pig, 12 pigs |  | ADRC’s | analysis software, | presence of rete | at the time of injury |
|  |  | 7.6 cm × | total |  | delivers as a | histology, | pegs, better | improves scarring |
|  |  | 7.6 cm, 2 mm |  |  | spray onto the | Immunohistochemistry, | vascularity, more | outcome |
|  |  | depth |  |  | wound | skin hardness via | normally |  |
|  |  |  |  |  | immediately | durometer | organised |  |
|  |  |  |  |  | post-injury vs |  | collagen. |  |
|  |  |  |  |  | untreated |  | Upregulation of |  |
|  |  |  |  |  | control |  | IL-6 expression |  |
|  |  |  |  |  |  |  | then |  |
|  |  |  |  |  |  |  | downregulation |  |
|  |  |  |  |  |  |  | in intervention |  |
|  |  |  |  |  |  |  | group |  |
| Jimi et al. | Clawn | Sharp excision | 4 on each | 15, 30, 60, | Use of the | Histological analysis, | Scar thickened | Chymase plays an |
| ([54](#_bookmark51)) | mini-pig | 7.5 cmx7.5 cm | pig, 8 pigs in | 90, 120, 150 | Clawn minipig | water content, chymase | up to 90 days | important role in |
|  |  | depth 0.15 cm | total |  | as a scarring | activity, | then decreased, | scar thickening |
|  |  |  |  |  | model |  | TGF-β1 greater | and Clawn pigs are |
|  |  |  |  |  |  |  | in scar, peaks at | a useful animal |
|  |  |  |  |  |  |  | day 15 then | model for HTS. |
|  |  |  |  |  |  |  | decreases, water |  |
|  |  |  |  |  |  |  | content peaks at |  |
|  |  |  |  |  |  |  | day 15 in scar, |  |
|  |  |  |  |  |  |  | chymase activity |  |
|  |  |  |  |  |  |  | rises in scar to |  |
|  |  |  |  |  |  |  | day 90 |  |
| Liu et al. | Bama | Electric | 4 | 60 (pressure | Pressure of | RNA analysis, gene | 568 DEG at 90 | Genes associated |
| ([49](#_bookmark46)) | mini-pig | dermatome, 8 × |  | application) | 3.4 kPa | expression, qPCR | days, 365 DEG | with transporter |
|  |  | 8 cm, 1.8 mm |  | 90, 120 |  |  | at 120 days, GO | activity and signal |
|  |  | deep |  |  |  |  | the DEG’s have | transducer activity |
|  |  |  |  |  |  |  | 50 functional | participate in the |
|  |  |  |  |  |  |  | categorical in | treatment of |
|  |  |  |  |  |  |  | cellular function. | pressure for HTS |
|  |  |  |  |  |  |  | α-SMA |  |
|  |  |  |  |  |  |  | decreased after |  |
|  |  |  |  |  |  |  | pressure therapy |  |
|  |  |  |  |  |  |  |  | (*continued*) |

TABLE A2 | Continued

| Study | Pig Breed | Scar Method | Wound number | Data collection point (days) | Intervention | Outcomes | Results | Authors comments |
| --- | --- | --- | --- | --- | --- | --- | --- | --- |
| Rodriguez- | Red | Branding iron, | 27 | 14, 21, 35 | Erg:YAG laser | mVSS, MSS, | Er:YAG laser | Model produces |
| Menocal | Duroc | 300°C, 12 s, |  |  | treatment | Histological | treated wounds | hypertrophic scar |
| et al. ([47](#_bookmark44)) |  | 27 mm |  |  | high, low vs | assessment, Western | had better | that mimics human |
|  |  | diameter |  |  | CO_2_ laser | blot, qPCR | scores in mVSS | burn scar. |
|  |  |  |  |  | high, low and |  | and MSS, low Er: |  |
|  |  |  |  |  | control (no |  | YAG best, |  |
|  |  |  |  |  | treatment) |  | Thinner dermis |  |
|  |  |  |  |  |  |  | In control, most |  |
|  |  |  |  |  |  |  | remodelling |  |
|  |  |  |  |  |  |  | observed is CO_2_ |  |
|  |  |  |  |  |  |  | laser |  |
|  |  |  |  |  |  |  | qPCR – Decorin |  |
|  |  |  |  |  |  |  | expression great |  |
|  |  |  |  |  |  |  | in both lasers on |  |
|  |  |  |  |  |  |  | low setting, |  |
|  |  |  |  |  |  |  | western blot – |  |
|  |  |  |  |  |  |  | MMP-9 increase |  |
|  |  |  |  |  |  |  | in ER;YAG at low |  |
|  |  |  |  |  |  |  | setting |  |
| Travis et al. | Red | Zimmer^®^ | 3 partial | 2, 4, 7, 9, | Presence of | Histological analysis, | Presence of | Biphasic presence |
| ([56](#_bookmark53)) | Duroc | dermatome 3″ | depth, 2 full | 11, 14, 28, | Fibrocytes in | immunohistochemistry, | LSP-1, CD45, | of ﬁbroblasts |
|  |  | × 3″ depth | depth on | 35, 42, 56, | healing skin |  | procollagen-1, | initially at acute |
|  |  | 0.06″ or 0.03″ and 4″ × 4″  depth 0.09″ | each pig (number of  pigs not | 70, 113 |  |  | increase  ﬁbrocyte staining from day 56, | response to healing and then a  second peak |
|  |  |  | given) |  |  |  | upregulation of | during remodelling |
|  |  |  |  |  |  |  | COL1A1 from |  |
|  |  |  |  |  |  |  | day 56 |  |
| Yun et al. | Red | Excision | 36 on each | ADSC given | Adipose | Scar photography and | Experimental | ADSC produce |
| ([57](#_bookmark54)) | Duroc | 3 cmx3 cm full | pig, 2 pigs | 50 days | derived stem | image analysis, | group surface | signiﬁcantly |
|  |  | thickness | total | post injury, | cells (ADSC) | Histological analysis, | area smaller, | smaller scars that |
|  |  | excision, |  | 0, 10, 20, |  | qPCR | colour and | appear more |
|  |  |  |  | 30, 40, 50 |  |  | pliability closer | similar to normal |
|  |  |  |  | days post |  |  | to normal skin. | skin. Work by |
|  |  |  |  | ADSC |  |  | Experimental | reducing mast cell |
|  |  |  |  |  |  |  | reduced | and myoﬁbroblast |
|  |  |  |  |  |  |  | ﬁbroblasts, and | activity. |
|  |  |  |  |  |  |  | suppression of |  |
|  |  |  |  |  |  |  | TGF-β1 |  |
| Yun et al. | Red | 3×3 cm^2^ sharp | 36 | 50, 60 (10 | Injection of | Photographic analysis, | Relaxin treated | Relaxin may have a |
| ([48](#_bookmark45)) | Duroc | excision – full |  | days post | relaxin in | histological analysis, | group had | prominent role in |
|  |  | thickness |  | intervention) | aliginate gel- | immunohistochemistry, | decreased scar | scar remodelling |
|  |  |  |  |  | encaspulated | qPCR | size, colour |  |
|  |  |  |  |  | virus in |  | index, pliability, |  |
|  |  |  |  |  | established |  | reduced MMP-1 |  |
|  |  |  |  |  | HTS |  | inhibitor and α- |  |
|  |  |  |  |  |  |  | SMA, |  |
|  |  |  |  |  |  |  | downregulated |  |
|  |  |  |  |  |  |  | TGF-β1, |  |
|  |  |  |  |  |  |  | upregulated |  |
|  |  |  |  |  |  |  | TGF-β3 |  |
| Zhu et al. | Red | Padgett^®^ | 41, 6 pigs | Weekly to | Assess | Macroscopic analysis, | Increased | Further study |
| ([60](#_bookmark57)) | Duroc | dermatome | total | 20 weeks | methodology | histological analysis, | expression of | required to see if |
|  |  | 8 cm × 8 cm, |  |  | for creating | immunhistochemistry | IGF-1, reduced | Red Duroc pig |
|  |  | depth 0.015 to |  |  | HTS |  | expression of | acceptable as a |
|  |  | 0.12″, 0.015″ |  |  |  |  | TGF-β1 in | human model for |
|  |  | intervals |  |  |  |  | deeper injuries. | HTS |
|  |  |  |  |  |  |  | Histologically |  |
|  |  |  |  |  |  |  | similar |  |
|  |  |  |  |  |  |  |  | (*continued*) |

TABLE A2 | Continued

| Study | Pig Breed | Scar Method | Wound number | Data collection point (days) | Intervention | Outcomes | Results | Authors comments |
| --- | --- | --- | --- | --- | --- | --- | --- | --- |
|  |  |  |  |  |  |  | appearance to |  |
|  |  |  |  |  |  |  | human HTS at |  |
|  |  |  |  |  |  |  | deeper injury. |  |
| Zhu et al. | Red | Padgett^®^ | 8 wounds | 10, 30, 60, | Evaluate TGF- | Histological analysis, | Increased | Findings correlate |
| ([62](#_bookmark59)) | Duroc | dermatome | on each pig, | 90 and 150 | β1, IFG-1, | immunohistochemistry, | expression of | with literature on |
|  |  | 7 cm × 7 cm, | 2 pigs total | post- | decorin and | qPCR | TGF-β1, IFG-1, | human HTS tissue |
|  |  | depth 0.015″, 0.030″, 0.045″  and 0.06″ |  | wounding | versican expression in porcine HTS |  | decorin reduced in deeper wounds. |  |

*Abberviations: α-SMA, alpha smooth muscle actin; ADSC, adipose derived stem cells; ADRC, adipose derived regenerative cells; COL1A1, Collagen type 1 Alpha 1 gene; DEG, Differentially expressed genes; Erg:Yag, erbium-doped yttrium aluminium garnet laser; GO, Gene ontology; HTS, Hypertrophic Scar; IGF-1, Insulin like growth factor -1; LSP-1, Lymphocyte speciﬁc protein - 1; MSS, Manchester scar scale; MMP, Matrix metalloproteinases; mVSS, modiﬁed Vancouver scar scale; PDL, Pulsed dye laser; qPCR, Quantitative Polymerase chain reaction; STSG, split thickness skin graft; TEWL, Transepidermal water loss; TGF, Transforming growth factor; VSS, Vancouver Scar Scale.*

TABLE A3 | Results from murine human skin graft models.

| Study | Murine type | Scar method | Data collection point (days) | Intervention | Outcomes | Results | Authors comments |
| --- | --- | --- | --- | --- | --- | --- | --- |
| Ding et al. | Nu/nu | X | X | X | X | X | Description of protocols from |
| ([69](#_bookmark66)) | mouse |  |  |  |  |  | Momtazi et al and Wang et al |
| Momtazi | Nu/Nu | 2.0 cm × 1.5 cm | 30, 60, 120, | Comparison of | MSS, histological | MSS score 15.9 ± | Model creates viable tissue |
| et al. ([67](#_bookmark64)) | mouse | sharp excision | 180 | xenograft with | analysis, | 0.2 xenograft vs 8.2 ± | that is morphologically, |
|  |  | + Human |  | human HTS | immunohistochemistry | 0.1 autograft control, | histologically and |
|  |  | Xenograft |  | tissue. |  | α-SMA present in xenograft | immunhistochemically identical to human HTS |
| Wang | Nu/nu | 2.0 cm × 1.5 cm | 0, 28, 56, | FTSG human vs | Macroscopic analysis, | Scar hypertrophy | Human skin grafted onto |
| et al. ([68](#_bookmark65)) | mouse | sharp excision | 112, 196 | STSG (autograft) | histological analysis, | develops over 4 | nude mice develops ﬁbrotic |
|  |  | + Human Xenograft |  | vs FTSH (autograft) | Immunohistochemistry | months, upregulation COL1α1, TGF-β1,  CTGF, More | scars which resemble HTS.  STSG ﬁbrose more than FTSG |
|  |  |  |  |  |  | macrophages and |  |
|  |  |  |  |  |  | mast cells in STSG |  |
| Zeplin | Nu/nu | 1.5 cm × 1.5 cm | 84 | Halofuginone- | qPCR | Treatment group | Halofuginon-eluting hybrid |
| et al. ([70](#_bookmark67)) | mouse | sharp excision |  | eluting SGS vs |  | reduced expression of | surface SGS increase anti- |
|  |  | + Human  Xenograft - |  | regular SGS |  | TGF-β1, COLA1A1,  CTGF, FGF2, MMP-2 | scarring effect by normalising  inﬂammatory scar gene |
|  |  | additional |  |  |  | and 9 | expression |
|  |  | contact burn |  |  |  |  |  |
|  |  | 80°C for 10 s |  |  |  |  |  |

*Abbreviations: α-SMA, alpha smooth muscle actin; COL1A1, Collagen type 1 Alpha 1 gene; CTGF, connective tissue growth factor; FGF2, Fibroblast growth factor 2 gene; FTSG, Full thickness skin graft; HTS, Hypertrophic Scar; MMP, Matrix metalloproteinases; MSS, Manchester scar scale; qPCR, Quantitative polymerase chain reaction; SGS, Silicone gel sheet; STSG, Split thickness skin graft; TGF, Transforming growth factor*.

TABLE A4 | Results from murine cultured human keloid models.

| Study | Murine type | Delivery method of keloid tissue | Data collection point (days) | Outcomes | Results | Authors comments |
| --- | --- | --- | --- | --- | --- | --- |
| Lee | Nu/J | Cultured human keloid | 14, 28, 42, | Survival rate, histological | 90% survival rate at 4 weeks. | Keloid cultures take on |
| et al. | athymic | tissue *in vivo* – | 56, 70, 84, | analysis, qPCR, Macroscopic | Elevated Col1A1, PAI-1, uPAR | athymic mice. |
| ([73](#_bookmark70)) | mouse | transplanted onto the | 98, 112 | analysis, Immunoﬂuoresence | elevated in keloid, keloid | Opportunity to |
|  |  | dorsum |  |  | implants larger at 8 weeks than | genetically manipulate |
|  |  | Normal human skin |  |  | control, GFP tagged human | cells used to make the |
|  |  | implants cultured as |  |  | keloid cells bifringence | graft |
|  |  | control |  |  | observed at 16 weeks |  |
| Shang | Nu/Nu | Subcutaneous injection of | 12, 28, 42, | Histological analysis, | Whole dermal keloid tissue | Easily reproducible |
| et al. | mouse | cultured human dermal | 84 | macroscopic analysis, cytokine | grafts more like human keloid | model to study human |
| ([74](#_bookmark71)) |  | keloid whole tissue vs |  | analysis | scar compared to keloid | keloid tissue. |
|  |  | cultured human keloid |  |  | ﬁbroblast only grafts |  |
|  |  | ﬁbroblasts only |  |  |  |  |
| Supp | Nu/Nu | GAG scaffold | 0, 14, 28, 42, | Histological analysis, | COL1A1, TGF-β1, POSN, | Keloid grafts survive |
| et al. | mouse | impregenated with cells: | 56, 70, 84 | immunohistochemistry, real | PAI2, FST, expression highest | and could an easily |
| ([71](#_bookmark68)) |  | K + F vs K + DKF vs K + |  | time qPCR | in DKF, followed by SKF. No | scalable model. |
|  |  | SKF vs KK + F vs KK + |  |  | differences observed in |  |
|  |  | DKF |  |  | COL1A1 expression. |  |
|  |  | Grafted onto mouse |  |  | Thick, disorganised collagen |  |
|  |  |  |  |  | bundles observed in DKF or |  |
|  |  |  |  |  | SKF containing grafts |  |
| Wang | BALB/c | PLGA scaffold cultured | 30, 60, 120, | Volume analysis, histological | Volume of keloid scaffold 12 × | Human keloid PLGA |
| et al. | athymic | with keloid ﬁbroblasts – | 180 | analysis, | 10 × 2 mm^3^ vs control scaffold | scaffold model could be |
| ([72](#_bookmark69)) | mouse | implanted into |  | immunohistochemistry | 2 × 2 × 0.4 mm^3^ | used to study ﬁbroblast |
|  |  | subcutaneous pocket |  |  | Presence of keloid ﬁbroblasts | function and effect of |
|  |  |  |  |  | in scaffold at day 180. | drugs |
|  |  |  |  |  | Formation of collagen spindles |  |

*Abbreviations: COL1A1, Collagen type 1 Alpha 1 gene; DKF, deep keloid ﬁbroblasts; F, normal ﬁbroblasts; FST, Follistatin gene; GAG, Glycosaminoglycan; GFP, Green ﬂuorescence protein; K, normal keratinocytes; KK, keloid keratinocytes; PAI2, plasminogen activator inhibitor gene; PLGA, Polylactic-co-gylcolic acid; POSN, Periostin gene; qPCR, Quantitative polymerase chain reaction; SKF, superﬁcial keloid ﬁbroblasts; TGF, Transforming growth factor; uPAR, Urokinase receptor*.

TABLE A5 | Results from murine whole keloid tissue graft models.

| Study | Murine type | Delivery method of keloid tissue | Data collection point (days) | Intervention | Outcomes | Results | Authors comments |
| --- | --- | --- | --- | --- | --- | --- | --- |
| Chen et al. ([80](#_bookmark77)) | BALB/c- | Whole | 7, 14, 28 | No injection | Histological analysis, | Combination group | Potential for |
|  | nu | transplanted |  | control vs BTXA | ﬁbroblast proliferation, | greatest reduction in | combined BTXA |
|  | mouse | human keloid |  | vs TCA vs TCA + | immunohistochemistry, | weight, and greatest | and TCA |
|  |  | tissue graft into |  | BTXA | scar weight | reduction in ﬁbroblast | intralesional |
|  |  | subcutaneous |  |  |  | proliferation | therapy in treating |
|  |  | space |  |  |  |  | HTS |
| Fanous et al. | Nu/nu | Whole | 7, 14, 28 | Saline control vs | Histological analysis, scar | Treatment groups had | Botox has a role |
| ([81](#_bookmark78)) | mouse | transplanted |  | BTXA vs TCA | weight | reduced weight | in HTS prevention |
|  |  | human keloid |  |  |  | BTXA group better |  |
|  |  | tissue graft into |  |  |  | organised collagen |  |
|  |  | subcutaneous |  |  |  |  |  |
|  |  | space |  |  |  |  |  |
| Philandrianos | Nu/nu | Full thickness | 0, 28, 56, 84, | No treatment | Macropscopic and | No differences | Further studies |
| et al. ([79](#_bookmark76)) | mouse | 8 mm human | 112 (all 4 | control vs LASER | Histological analysis for | observed between | required to |
|  |  | keloid tissue | weeks post | treatment, vs | heatshock protein | scars | determine effect |
|  |  | surgically sutured | graft) | 4 mm resection |  |  | of LASER on |
|  |  | to mouse |  | vs 4 mm |  |  | keloid |
|  |  |  |  | resection + |  |  |  |
|  |  |  |  | LASER |  |  |  |
| Qiu et al. ([78](#_bookmark75)) | BALB/c- | Whole | 7, 14 | P144^®^ topical vs | Histological analysis | Reduction in collagen I | P144^®^ may have |
|  | nu  mouse | transplanted  human keloid |  | topical placebo |  | and III expression in  P144^®^ group | future  applications but |
|  |  | tissue graft into |  |  |  |  | further research |
|  |  | subcutaneous |  |  |  |  | required |
|  |  | space |  |  |  |  |  |
| Yang et al. ([75](#_bookmark72)) | BALB/c- | Whole | 7, 14, 28 | Saline control vs | Fibroblast proliferation, | Combination | Combination |
|  | nu | transplanted |  | Verapamil vs | Immunohistochemistry, | decreased ﬁbroblast | therapy more |
|  | mouse | human keloid |  | Verapamil + TCA | scar weight | proliferation, scar | effective in |
|  |  | tissue graft into |  | intralesional |  | weight and increased | reducing scar – |
|  |  | subcutaneous |  | injections |  | decorin expression | more work |
|  |  | space |  |  |  |  | needed |
| Yang et al. ([76](#_bookmark73)) | BALB/c- | Whole | 7, 14, 28 | Saline control vs | Fibroblast proliferation, | Fibroblast proliferation | Combination of |
|  | nu | transplanted |  | TCA vs TCA + | histological analysis, scar | least in TCA group, | TCA, verapamil |
|  | mouse | human keloid |  | verapamil + IFN | weight | combination group | and IFN effective |
|  |  | tissue graft into |  |  |  | less disorganised | in reducing HTS |
|  |  | subcutaneous |  |  |  | collagen and smallest | tissue. |
|  |  | space |  |  |  | scar in size and weight |  |
| Yang et al. ([77](#_bookmark74)) | BALB/c- | Whole | 7, 14, 28 | No injection | qPCR | INFα2b group | Suggest maintain |
|  | nu | transplanted |  | control vs TCA vs |  | strongest expression | dose of INFα2b |
|  | mouse | human keloid |  | verapamil vs |  | of decorin and | along with high |
|  |  | tissue graft into |  | IFNα2b |  | MMP13, TCA stronger | dose verapamil to |
|  |  | subcutaneous |  |  |  | expression of decorin | improve HTS |
|  |  | space |  |  |  | compared to |  |
|  |  |  |  |  |  | verapamil, stronger |  |
|  |  |  |  |  |  | expression of MMP13 |  |

*Abbreviations: BTXA, Botulinum toxin A; HTS, Hypertrophic scar; INF, interferon; MMP, Matrix metalloproteinases; qPCR, Quantitative polymerase chain reaction; TCA, triamcinolone acetonide.*

TABLE A6 | Results from murine thermal injury models.

| Study | Murine type | Thermal injury method | Data collection point (days) | Intervention | Outcomes | Results | Authors comments |
| --- | --- | --- | --- | --- | --- | --- | --- |
| Ibrahim | Nu/nu mouse | Thermal injury | 3, 7, 9, 11, | Burn only | Macroscopic analysis, | Skin grafts | Resulting scar was |
| et al. |  | brass rod, 100°C | 14, 28, 70, |  | histological analysis, | contracted, | different to human |
| ([83](#_bookmark80)) |  | for 1 s, followed | 168 |  | immunohistochemistry | increased | hypertrophic scar |
|  |  | by excision at 3 |  |  |  | vascularity, | tissue. |
|  |  | days at auto |  |  |  | macrophages and |  |
|  |  | FTSG |  |  |  | mast cells |  |
| Lorden | C57BL/6 mouse | Thermal burn | 7, 14, 30 | Effect of | Histological analysis, | Scaffold group | Collagen coated |
| et al. |  | unspeciﬁed, |  | microﬁbrous | biomechanics, | improved collagen | scaffold may have |
| ([84](#_bookmark81)) |  | excised 3 days |  | scaffold in | macroscopic | arrangement, | merit in preventing |
|  |  | later |  | mitigating HTS | appearance | reduced | HTS |
|  |  |  |  | contraction |  | contraction and |  |
|  |  |  |  |  |  | stronger scar |  |
| Lu et al. | Immunocompetent | Submersion into | 15 | CL injection | Histological analysis, | Control scars | CL may be suitable |
| ([85](#_bookmark82)) | mouse | 100°C water for |  | subcutaneous vs | qPCR | developed | treatment for HTS |
|  |  | 8s |  | intranperitoneal vs |  | collagen whorl |  |
|  |  |  |  | no intervention |  | patterns, less in CL |  |
|  |  |  |  | control |  | groups, reduced |  |
|  |  |  |  |  |  | TGF-β1 expression in CL |  |
| Zeplin | Nu/nu mouse | 1.5 cm × 1.5 cm | 84 | Halofuginone- | qPCR | Treatment group | Halofuginon- |
| et al. |  | sharp excision + |  | eluting SGS vs |  | reduced | eluting hybrid |
| ([70](#_bookmark67)) |  | Human |  | regular SGS |  | expression of TGF- | surface SGS |
|  |  | Xenograft -  additional |  |  |  | β1, COLA1A1,  CTGF, FGF2, | increase anti-  scarring effect by |
|  |  | contact burn 80°  C for 10 s |  |  |  | MMP-2 and 9 | normalising  inﬂammatory scar |
|  |  |  |  |  |  |  | gene expression |

*Abbreviations: COL1A1, Collagen type 1 Alpha 1 gene; CL, Clondronate liposome; CTGF, connective tissue growth factor; FGF2, Fibroblast growth factor 2 gene; HTS, Hypretrophic scar; MMP, Matrix metalloproteinases; qPCR, Quantitative polymerase chain reaction; SGS, Silicone gel sheet; TGF, Transforming growth factor.*

TABLE A7 | Results from murine incision and stretch models.

| Study | Murine type | Scar method | Data collection point (days) | Intervention | Outcomes | Results | Authors comments |
| --- | --- | --- | --- | --- | --- | --- | --- |
| Murphy | Immunocompetent | Full thickness | Every 2 days | Oral losartan via | Histological | Losartan treated rat had | Losartan has |
| et al. ([87](#_bookmark84)) | mouse | excision followed | until 28 days | water vs normal | analysis, qPCR, | smaller scars and | potential as a |
|  |  | by mechanical  loading of wound |  | water control | macroscopic  appearance | reduced α-SMA and  CD68 expression | novel therapy for  preventing HTS |
|  |  | edges |  |  |  |  |  |
| Shan | Immunocompetent | Full thickness | 0, 10, 14 | No treatment | Histological | Naringenin inhibits | Naringenin could |
| et al. ([88](#_bookmark85)) | mouse | excision followed  by mechanical |  | control vs 25 µM | analysis, qPCR,  Western blot | ﬁbroblast activation,  reduced inﬂammation | have use a scar  treatment |
|  |  | loading of wound |  |  |  | Reduced expression of |  |
|  |  | edges |  |  |  | IL-1β, IL-6, TGF-β1+ TNF-α |  |
| Zhou | Immunocompetent | Excision of rat tail | 0, 2, 10, 16, | 3×3 mm vs 6 × | Histological | No wound contraction in | Future HTS |
| et al. ([9](#_bookmark7)) | rat | skin + application | 20,42, 84, | 6 mm vs 9 × | analysis, qPCR, | tail wounds, high strain | model, more cost |
|  |  | of steel ring to | 168 | 9 mm, each with | macroscopic | wounds produced thicker | effective, faster |
|  |  | add strain  Dorsal skin injury |  | no, little or high  strain | appearance | HTS. TGF-β1 and α-SMA  expression equivocal to | and easily  reproducible |
|  |  | as control |  |  |  | human HTS |  |

*Abbreviations: α-SMA, alpha smooth muscle actin; HTS, hypertrophic scar; IL, Interleukin; qPCR, Quantitative polymerase chain reaction; TGF, Transforming growth factor.*

TABLE A8 | Results from murine biopsy and antibiotic models.

| Study | Murine type | Scar method | Data collection point (days) | Intervention | Outcomes | Results | Authors comments |
| --- | --- | --- | --- | --- | --- | --- | --- |
| Cameron | Immunocompetent | Subcutaneous | 0, 28, 56 | 28 day bleomycin | Histological analysis, | Bleomycin group | Potential model for |
| et al. ([91](#_bookmark88)) | mice | infusion of |  | infusion vs | qPCR | thicker dermis, | HTS, avoids |
|  |  | bleomycin |  | control Saline at |  | thinner dermis – | contraction |
|  |  |  |  | constant rates |  | akin to human HTS. | created by |
|  |  |  |  |  |  | Increased | panniculus |
|  |  |  |  |  |  | expression of | carnosus injury |
|  |  |  |  |  |  | TGF-β1 |  |
| Sahin | Immunocompetent | Full thickness | 0, 10, 30 | Topical heparin | Histological analysis, | Contractubex^®^ | Contractubex^®^ |
| et al. ([89](#_bookmark86)) | rats | skin biopsy –  size not given |  | vs alantoin vs  Contractubex^®^ vs | Immunohistochemistry,  TEM | group thinnest  epidermis, | improves quality of  wound healing and |
|  |  |  |  | control |  | completely | resulting scar |
|  |  |  |  |  |  | keratinised on TEM |  |

*Abbreviations: HTS, Hypertrophic scar; qPCR, Quantitative polymerase chain reaction; TEM, Transmission electron microscopy; TGF, Transforming growth factor.*

TABLE A9 | Results from other animal models.

| Study | Animal | Scar method | Data collection point (days) | Intervention | Outcomes | Results | Authors comments |
| --- | --- | --- | --- | --- | --- | --- | --- |
| Kimura | Mexican | 3×3 cm^2^ full | 0, 30, 90 | X | Macroscopic | Macroscopic | Mexican hairless |
| et al. ([94](#_bookmark91)) | hairless | thickness |  |  | appearance, | pigmented HTS, well- | dog may be a |
|  | dog | excision |  |  | histological | organised collagen | suitable alternative |
|  |  |  |  |  | analysis | present on histology | model for HTS |
| Igarashi | Primate | 2 cm long full | 0, 35, 42 | Control vs GB1101 | Macroscopic | Treated scars had a | GB1101 may have a |
| et al. ([93](#_bookmark90)) | Marmoset | thickness |  | injection prior to incision | appearance, | thinner epidermis and | role in preventing |
|  |  | abdominal incision |  | vs GB1101 topical post- incision (once only) | histological analysis | dermis with a resulting  ﬂatter scar | HTS in surgical patients. |

*Abbreviations: GB1101, pyrrole-imidazole polyamide; HTS, Hypertrophic scar.*

TABLE A10 | Results from human *in vivo* models.

| Study | Study design | Patient group | Intervention | Data collection points | Outcomes | Results | Author comments |
| --- | --- | --- | --- | --- | --- | --- | --- |
| Cruz- | RCT | Patients post bilateral | SGS vs No treatment | Dressing worn for | Clinical assessment | Untreated scars; | SGS helps reduced |
| Korchin |  | McKissock reduction |  | 2 months – follow | of scar hypertrophy | 45% ﬂat scars, | the presence of HTS |
| et al. ([95](#_bookmark92)) |  | mammaplasty |  | up at 6 months |  | HTS |  |
|  |  |  |  |  |  | Silicone group;  75% ﬂat scars and |  |
|  |  |  |  |  |  | 25% HTS |  |
| Dunkin | Clinical | 113 healthy | Controlled | 1, 2, 3, 4, 6, 10, | Scar surface area, | 36 weeks – mean | Jig is a well-tolerated |
| et al. ([100](#_bookmark97)) | study | volunteers | standardised scratch | 18, 24, 36 weeks | scar thickness | scar length of | standardised method |
|  |  |  | injury on hip skin to | post-injury | (USS), histological | 34.9 ± 1.0 mm | of creating scar. |
|  |  |  | determine depth at |  | analysis | (68% of the |  |
|  |  |  | which scar occurs |  |  | original wound |  |
|  |  |  |  |  |  | length) |  |
|  |  |  |  |  |  | Visible scar |  |
|  |  |  |  |  |  | formed from |  |
|  |  |  |  |  |  | 0.56 ± 0.03 mm or |  |
|  |  |  |  |  |  | 33.1% depth |  |
|  |  |  |  |  |  | injury |  |
| Kong | RCT | Knee replacement | Liquid silicone gel vs | Gel applied 5 days | VSS | Thinner and lighter | Silicone gel has no |
| et al. ([98](#_bookmark95)) |  | patients | placebo gel | post op daily for 1 |  | scars in treatment | beneﬁcial effect on |
|  |  |  |  | month, follow up |  | group, no other | scar pain and itch |
|  |  |  |  | 3, 6 and 12 |  | difference | during post-op |
|  |  |  |  | months |  |  | period |
| Lanier | Clinical | Abdominoplasty | 20 full thickness | Left to heal for 12 | - | - | Model offers a |
| et al. ([101](#_bookmark98)) | study | patients – skin | incisions over | weeks |  |  | unique, cost- |
|  |  | planned for excision | abdomen - 2 cm in |  |  |  | effective method for |
|  |  |  | length - sutured |  |  |  | assessing scar |
|  |  |  |  |  |  |  | treatments |
| Niessen | RCT | Patients undergoing | SGS vs Micropore^®^ | Dressings worn | Clinician | 3 months 64.3% | SGS may in fact |
| et al. ([96](#_bookmark93)) |  | bilateral breast | tape | for 3 months, | assessment of scar | of patients at least | increase risk of HTS |
|  |  | reduction |  | follow up at 3,6 | height and width, | 1 HTS |  |
|  |  |  |  | and 12 months | Patient opinion of | 6 months – 29 |  |
|  |  |  |  |  | itch and pain | HTS in SGS vs 13 |  |
|  |  |  |  |  |  | control |  |
|  |  |  |  |  |  | 12 months – 19 |  |
|  |  |  |  |  |  | HTS in SGS vs 7 |  |
|  |  |  |  |  |  | control |  |
| Sproat | RCT | Established | 1 TCA injection vs | 12 weeks | Patient preference | 11/14 preferred | SGS is preferred |
| et al. ([99](#_bookmark96)) |  | symptomatic mid-line | SGS worn for 12 h/ |  | appearance of the | silicone, 2 | treatment by |
|  |  | sternotomy HTS | day for 12 vs no |  | scar, pain, itch and | preferred TCA, 1 | patients, better scar |
|  |  |  | treatment |  | ease of the | had no preference | and relief |
|  |  |  |  |  | treatment |  |  |

*Abbreviations: HTS, Hypertrophic scar; RCT, Randomised Controlled Trial; SGS, Silicone gel sheeting; TCA, triamcinolone acetonide; USS, Ultrasound Scanner; VSS, Vancouver Scar Scale*.

TABLE A11 | Results from human *in vitro* models.

| Study | Human tissue type | Scar method | Data collection point (days) | Intervention | Outcomes | Results/Authors comments |
| --- | --- | --- | --- | --- | --- | --- |
| Chawla  et al. ([103](#_bookmark100)) | HTS ﬁbroblast culture | 3D  collagen | X | Histological analysis, qPCR | Structure, histology and PC | In vitro created dermal scar showed α- SMA expression, excessive collagen and |
|  |  | structure |  |  |  | contraction like that of HTS. Model may |
|  |  |  |  |  |  | serve as an alternative for test scar |
|  |  |  |  |  |  | treatments. |
| Lee et al. | Human keloid | Spheres of | Injection with | Histological analysis, | Intervention group – | Immunohistochemistry proﬁle like that of |
| ([102](#_bookmark99)) | tissue | tissue | TCA | Immunohistochemistry | tissue regressed, | TCA treated keloid in human, |
|  |  | cultured |  |  | reduced expression of |  |
|  |  |  |  |  | collagen I, III and |  |
|  |  |  |  |  | elsatin |  |
| Reijnders | Immortalised | 3D bovine | Injured with | Histological analysis, | Morphology similar to | Useful as a model that does not rely on |
| et al. ([104](#_bookmark101)) | ﬁbroblasts and | collagen | heat and cold | Immunohistochemistry, | that of human skin, | fresh human tissue. |
|  | keratinocytes | matrix | injuries | TEM | has the ability heal |  |

*Abbreviations: α-SMA, alpha smooth muscle actin; HTS, Hypertrophic scar; qPCR, Quantitative polymerase chain reaction; TCA, triamcinolone acetonide; TEM, Transmission electron microscope.*
